# Integron Functionality and Genome Innovation: An Update on the Subtle and Smart Strategy of Integrase and Gene Cassette Expression Regulation

**DOI:** 10.3390/microorganisms10020224

**Published:** 2022-01-20

**Authors:** Érica L. Fonseca, Ana Carolina Vicente

**Affiliations:** Laboratório de Genética Molecular de Microrganismos, Instituto Oswaldo Cruz/FIOCRUZ, Avenida Brasil 4365, Manguinhos, Rio de Janeiro 21040-900, Brazil; anapaulo@ioc.fiocruz.br

**Keywords:** integron, transcription, translation, global regulator, SOS response, adaptation, transcription interference, translation coupling, translation attenuation, gene cassette expression

## Abstract

Integrons are considered hot spots for bacterial evolution, since these platforms allow one-step genomic innovation by capturing and expressing genes that provide advantageous novelties, such as antibiotic resistance. The acquisition and shuffling of gene cassettes featured by integrons enable the population to rapidly respond to changing selective pressures. However, in order to avoid deleterious effects and fitness burden, the integron activity must be tightly controlled, which happens in an elegant and elaborate fashion, as discussed in detail in the present review. Here, we aimed to provide an up-to-date overview of the complex regulatory networks that permeate the expression and functionality of integrons at both transcriptional and translational levels. It was possible to compile strong shreds of evidence clearly proving that these versatile platforms include functions other than acquiring and expressing gene cassettes. The well-balanced mechanism of integron expression is intricately related with environmental signals, host cell physiology, fitness, and survival, ultimately leading to adaptation on the demand.

## 1. Introduction

### Integron Functionality Is Directly Linked with Adaptation on Demand

**Figure 1:**
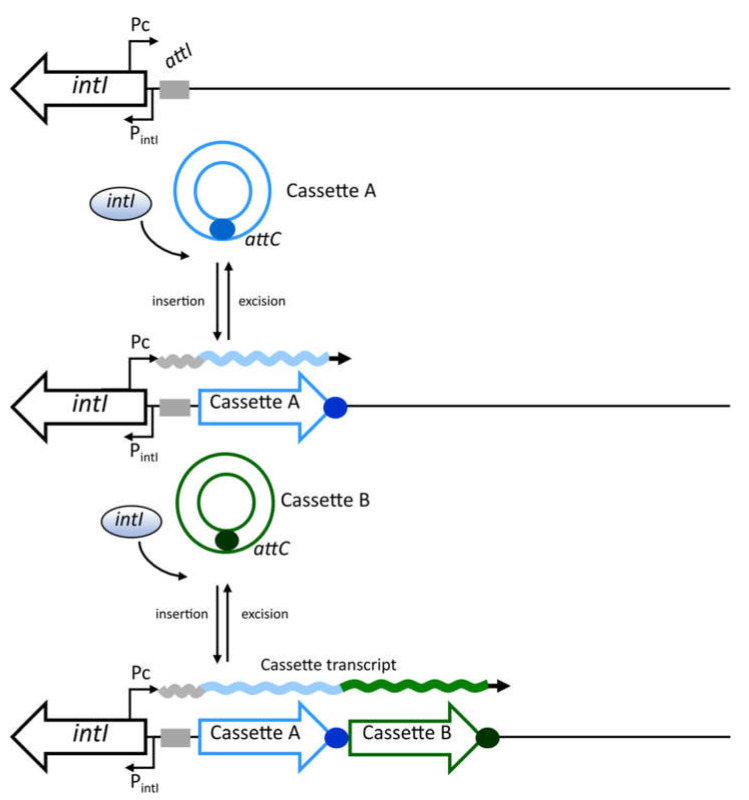
Schematic representation of integron structure and functionality. The integrase *intI*, the *attI* recombination site, and the P_intI_ and Pc promoters are showed. The gene cassettes and their cognate *attC* recombination sites are indicated by circles and arrows representing the free circular and integrated forms, respectively. The site-specific recombination events (integration and excision) mediated by IntI integrase are indicated. Cassette transcripts generated from the Pc promoter are indicated by curved lines.

Integrons are versatile genetic platforms involved with the acquisition, stockpiling, excision, and rearrangements of gene cassettes by site-specific recombination events mediated by integrase activity [1]. Cassettes are circular mobilizable structures comprising a gene bounded to a cassette-associated recombination site, called *attC*. In general, genes included in cassettes are devoid of promoter sequences due to the proximity of their initiation codons and the 5′ boundary of the cassette [2]. The basic structure of an integron comprises the *intI* integrase gene, a member of the tyrosine recombinase family, the *attI* recombination site, where cassettes are recombined, the P_intI_ integrase promoter, and a common promoter (Pc), generally embedded in *intI*, that drives the expression of promoterless cassette-associated genes, featuring the integrons as natural expression vectors [2,3] (Figure 1). The integration of a circular cassette intermediate preferentially occurs at the *attI* site (*attI* X *attC*), ensuring the immediate expression of the new integrated cassettes by the upstream Pc promoter. During cassette excision, the integrase catalyzes recombination between two adjacent *attC* sites (*attC* X *attC*) [4]. These site-specific reactions mediated by the integrase precisely occur between the first and second bases of the 7-bp core site sequence GTTRRRY shared among *attI* and *attC* sites (Figure 1) [5,6].

Several classes of integrons have been recognized based on integrase protein sequences, which shared 45–59% amino acid identity. Three major integron classes (classes 1, 2, and 3) are featured by their strong link with antibiotic resistance gene cassettes and the emergence of resistance phenotypes and, therefore, these elements have been considered resistance integrons (RIs). Moreover, due to their intimate association with mobile genetic elements, these integrons classes were also classified as mobile integrons (MIs), constituting the major vectors of antibiotic resistance spread among bacteria. The class 1 and 3 integrons are associated with Tn*402*-related transposons, while class 2 integrons are only found in association with the Tn*7* transposon module [7].

In spite of these common features, the RIs/MIs present some peculiarities. Among them, class 1 integrons are the most widespread and have been continuously involved with resistance emergence to a remarkable range of antibiotic treatments. Together with its 5′ conserved segment (CS), composed by the *intI1* integrase gene, the *attI1* recombination site, and the Pc and P_intI1_ promoters [2,3], the class 1 integrons also present a 3′CS. This region comprises the gene cassettes *qacEΔ1*, fused in its 3′ end with the *sul1*, which confer resistance to quaternary ammonium compounds and sulfonamides, respectively, followed by *orf5* of unknown function [1]. This class is also characterized by harboring a huge variety of resistance gene cassettes involved with resistance to non-related antibiotic classes, such as aminoglycosides, β-lactams, carbapenems, trimethoprim, chloramphenicol, rifampin, fluoroquinolones, macrolides, and streptomycin [1,5,7]. On the other hand, although class 2 integrons also harbor a typical 5′CS composed by the *intI2* gene, (whose predicted integrase shared 46% identity with IntI1), the *attI2*, and Pc and P_intI2_ promoters (see below Section 2.1.2 and Section 2.7), they carry a limited number of resistance gene cassettes [7]. The class 2 integrons normally include the *dfrA*, *aadA,* and *sat* gene cassettes, which confer resistance to trimethoprim, streptomycin, and streptothricin, respectively, and *orfX* of unknown function. Different from classes 1 and 2, the class 3 integrons are the rarest elements among the RIs/MIs. Despite class 3 integrons present a 5′CS backbone very similar to that found for class 1 integrons (see Section 2.1.3 and Section 2.8), this other class codes an integrase 59% identical to IntI1, which are able to recognize the same gene cassettes, and harbors an *attI3* longer than *attI1* (131 bp versus 65 bp).

Another integron category is known as chromosomal integrons (CIs), which have the same basic structure that defines an integron (*IntI*, *attI*, Pc, and P_intI_), but present several particularities relative to their mobile counterparts (MIs). Firstly, CIs are non-mobilizable platforms embedded in the chromosome of a large number of environmental Gram-negative bacteria [4]. These elements are greater in size relative to MIs and may comprise a significant portion of the genome. For example, the *Vibrio cholerae* superintegron (IntVchA), which is the best-studied CI [8], harbors around 180 cassettes. Besides the remarkable difference in size between MIs (dozens of cassettes) and CIs (hundreds of cassettes), another important feature of CIs is that most of the gene cassettes found in these platforms codes for unknown proteins, which accounts for the host genomic plasticity (for revision see reference [4]). Furthermore, unlike the resistance gene cassettes carried on RIs/MIs, which are associated with unique and unrelated *attC* sites, the recombination sites of CI cassettes are represented by almost identical repeats of approximately 120 bp that occur along the CI array flanking each gene. These repeats are species-specific in a way that they were named according to the organism where they were found, for example, the *V. cholerae* CI harbors the *V. cholerae* repeats (VCRs) as cassette recombination sites [8]. Additionally, the CIs have been pointed as the ancestors and the reservoirs of gene cassettes currently found in MIs [9]. In fact, a previous study demonstrated that the *qnrVC1* gene cassette, firstly identified in a class 1 integron, was not associated with a typical *attC*, but with the recombination site that characterizes the *V. parahaemolyticus* CI (*V. parahaemolyticus* repeats—VPRs) [10]. Afterward, Fonseca and Vicente [11] observed that several other *qnrVC* alleles found in mobile platforms were always linked to CI-borne recombination sites from different *Vibrio* species. This evidence strongly indicated that *qnrVC* was an archeological trait of CIs in the current MIs, corroborating the previous hypothesis that chromosomal integrons are the source of cassettes present in drug-resistance integrons [9]. The main differences among RIs/MIs are featured in Table 1.

The integrons are ubiquitous in nature and harbor an extraordinarily high diversity of gene cassettes. In fact, these platforms have been revealed as key elements of bacterial adaptation and genome evolution, playing a role that goes beyond that involved with the antibiotic resistance emergence [4,7,12,13,14]. Bacteria may take advantage of different mechanisms to bypass the antibiotic action. However, the emergence of antibiotic resistance is usually associated with biological burden leading to a fitness cost for the bacterial host [15]. The most prominent advantage of integrons for genome innovation and adaptive success lies in the efficient and elegant modulation of gene cassette and integrase expression [13,16], which is tightly coupled with bacterial cell physiology [13,17,18,19,20,21,22]. The subsequent integration events lead to an in tandem stacking of gene cassettes, of which only those closest to Pc promoter are expressed, while more distal cassettes remain silenced. Considering that gene cassettes can be independently mobilized by the integrase, the inserted cassette array can be modified by excisions, reassortments, and novel acquisition events [23]. In this case, silenced or new cassettes can be recruited to the first position, allowing bacteria to rapidly adapt to novel environmental challenges, such as multiple-antibiotic treatment regimes, without causing a fitness cost burden to the cell. Consequently, in a bacterial population harboring integrons with different cassette arrangements, any new variant will promptly express genes that could be involved with advantageous phenotypes, such as multidrug resistance [24]. Therefore, the variable and diverse cassette content of an integron reflects the retrospect of adaptive events and represents a reservoir of functions that can be recalled on demand [13].

This review aimed to provide an up-to-date overview of the fine-tuning regulation of integrase and gene cassette expression at both transcriptional and translational levels, and of the intricate relationship between integrons and cell physiology components that feature these platforms as powerful tools for genome innovation and bacterial adaptation.

## 2. Transcriptional Regulation in Integrons

The expression of gene cassettes inserted in integrons is under a tight and complex regulation, which may be modulated at both transcriptional and translational levels by different mechanisms. At means of transcription control, the following aspects influencing gene cassette expression will be discussed in detail below: the activity and strength of the common cassette promoter regions (Pc and P2); the position effect imposed by the physical distance of gene cassettes relative to Pc; the role of cassette-borne promoters and other additional transcriptional units. All of them may affect the amount of mRNA produced by each gene cassette inserted in the integron and, therefore, in the case of RIs, the antibiotic susceptibility of the host strains [3].

### 2.1. The Role of Pc Promoters on Gene Cassette Transcription

#### 2.1.1. Pc Promoters in Class 1 Integrons

As aforementioned, the gene cassettes captured by integrons are mostly devoid of promoter sequences, in a way that their transcription is driven by the Pc promoter embedded in the integron 5′ conserved segment (Figure 1) [3]. The Pc promoter is embedded in class 1 integrase gene, 223 to 252 bp from the *attI1* recombination crossover point [2,3]. The asymmetric nature of cassette recombination involving the folded bottom strand of the *attC* site leads to cassettes insertion in the correct orientation relative to Pc promoter [25]. The *intI1* sequences present more than 99% nucleotide conservation with differences mostly found within the Pc promoter region, indicating the existence of distinct Pc variants [2,3,26]. Such variants were defined based on polymorphisms in the canonical Pc promoter (named Pc Strong), which presents an identical –35 and highly conserved –10 hexamers relative to σ^70^ consensus promoter sequences [3].

Until now, 13 Pc and four P2 variants, as well as a diversity of Pc + P2 combinations, have been identified in naturally occurring integrons by in vitro and in silico analyses [2,3,26,27,28,29] (Table 2), and it can be expected that new ones will continually emerge by random point mutations in Pc promoter sequences. Indeed, new Pc variants arise in response to the need of expressing gene cassettes more efficiently due to stronger antibiotic selective pressure, leading to the selection of more efficient Pc sequences.

The more frequently identified Pc variants in both clinical and natural environments are the Pc Strong (PcS), Pc Weak (PcW), Pc Hybrid 1 (PcH1), and Pc Hybrid 2 (PcH2) [2,3,27,30,31,32,33,34]. They were the first Pc variants characterized and their classification was based on the relative transcription strengths, as detailed below. The PcW variant presents polymorphisms in both –35 and –10 hexamers relative to the canonical PcS, while PcH1 and PcH2 contain the –35 and –10 hexamers of PcW and PcS in inverse combinations (PcH1: –35 of PcW and –10 of PcS; PcH2: –35 of PcS and –10 of PcW) (Table 2). Although the unique difference between PcH1 and PcS is one nucleotide in the –35 region, a remarkable difference in their transcription strength was observed (Table 2) [3,28]. These findings indicate the relevance of the nature and position of polymorphisms in the determination of promoter activity, contributing significantly to variations in transcription strengths.

Other Pc configurations have been identified, but in very low frequencies, such as PcIn42, PcIn116, PcPUO, reported in the class 1 integrons In42, In116 and in plasmid PUO901, respectively, and the Super Strong Pc (PcSS) [26,35,36,37,38]. The PcSS emerged due to a C to T replacement of the fifth base of the PcS –35 sequence. This unique point mutation led to a remarkable increase in the expression of the integron-associated *bla*_IMP-5_ gene cassette and on imipenem resistance, suggesting that PcSS was significantly stronger than PcS [38].

**Table 2:** Genetic and functional features of Pc and P2 variants and the different Pc + P2 combinations in class 1 integrons.

Pc Variant ^1^	–35 Sequence	Spacer Region (bp)	–10 Sequence	Transcription Strength ^2^	Reference
Pc variants with polymorphisms inside –35 and –10 regions					
PcS	TTGACA	17	TAAACT	1240	[2,3,26,28]
PcH2	TTGACA	17	TAAGCT	840	[26,27,28]
PcH1	TGGACA	17	TAAACT	220	[2,3,26,28]
PcSS	TTGATA	17	TAAACT	100	[26,38]
PcPUO	TCGACA	17	TAAACT	60	[26,35]
PcW	TGGACA	17	TAAGCT	40	[2,3,26,28]
PcIn42	TTGGCA	17	TAAACT	40	[26,36]
PcIn116	TTGACA	17	TGAACT	10	[26,37]
Pc variants with polymorphisms upstream the –10 region					
PcS_TGN–10_	TTGACA	17	(TGN)TAAACT	1160	[26]
PcH2_TGN–10_	TTGACA	17	(TGN)TAAGCT	860	[26]
PcW_TGN–10_	TGGACA	17	(TGN)TAAGCT	720	[26,39]
PcH1_TTN–10_	TGGACA	17	(TTN)TAAACT	375	[26]
PcW_TAN–10_	TGGACA	17	(TAN)TAAGCT	undetectable	[26]
Pc variants combined with P2					
PcS + P2	TTGACA TTGTTA	17 17	TAAACT TACAGT	1000	[26,28]
PcW_TGN–10_ + P2	TGGACA TTGTTA	17 17	(TGN)TAAGCT TACAGT	620	[26]
PcH1 + P2	TGGACA TTGTTA	17 17	TAAACT TACAGT	380	[26,28]
PcW + P2	TGGACA TTGTTA	17 17	TAAGCT TACAGT	340	[2,3,26,28]
Pc variants combined with P2 variants					
PcW_TGN–10_ + P2m1	TGGACA TTGTTA	17 17	(TGN)TAAGCT GACAGT	NP	[26]
PcH1 + P2m1	TGGACA TTGTTA	17 17	TAAACT GACAGT	NP	[26]
PcW + P2m2	TGGACA TTGTTA	17 17	TAAGCT TACACA	NP	[26]
PcW + P2m3	TGGACA TTGTTA	17 17	TAAGCT TACAAT	720	[29]

^1^ Underlined bases represent the polymorphisms relative to σ^70^ consensus promoter from *Escherichia coli* –35 TTGACA (16–18 bp) –10 TATAAT [3]. ^2^ Relative transcription strengths values were estimated by measuring the β-galactosidase activity of Pc-*lacZ* transcriptional fusions as reported in reference [26]. NP, not performed.

Nucleotide modifications other than those found in –35 and –10 hexamers of Pc also contribute to variations in transcription efficiencies. A previous study demonstrated that the occurrence of a C/G mutation close to the –10 hexamer of PcW (2 bp upstream) was able to increase transcription of inserted gene cassettes. This mutation extended the –10 motif (TGN), which enhances transcription efficiency of *E. coli* σ^70^ promoters, creating a new promoter variant named PcW_TGN-10_ [26,39,40]. Although rare, such TGN-10 motif has also been found in association with PcS and PcH2 [26,34]. Two other Pc variants were raised from additional mutations upstream –10 hexamer. They were designated PcW_TAN-10_ and PcH1_TTN-10_, which presented the C/A and C/T mutations, respectively, 2bp upstream –10 region, and both variants seem to be extremely rare [26,40].

Additionally to Pc, a second promoter, named P2, is found 119 bp downstream from Pc in all integrons (Table 2), however, in most cases, this promoter is inactive due to a short spacer region (14 bp instead of 17 bp). In some integrons, insertion of three G residues increases the P2 spacer region from 14 to 17 bp, generating a functional promoter [3]. This active P2 is almost exclusively found in association with PcW (PcW + P2) and accounts for 90% of transcription when this version is present [2,3]. More recent studies revealed that PcW + P2, PcW_TGN-10,_ and PcW_TGN-10_ + P2 variants occurred in PcW-containing integrons much more frequently than PcW alone [26,29,34]. The active P2 had also been rarely identified in association with PcH1 and PcS [28]. Additionally, three rare mutated forms of active P2, named P2m1, P2m2, and P2m3, were reported in association with PcW and PcH1 [26,29]. A fourth P2 variant, named here P2m4, was found in a defective integron harboring a truncated integrase gene. This promoter presented a polymorphism in the first position of –10 hexamer relative to the canonical P2 (Table 2). This truncation in *intI1* led to Pc deletion, in a way that P2m4 was the unique common promoter in that integron and seemed to control gene cassette expression [41].

So far, dozens of these Pc variants and Pc + P2 combinations had their activity experimentally determined (Table 2). It was revealed distinct relative transcription strengths that directly affect the expression of resistance gene cassettes inserted in class 1 integrons and, consequently, the level of antibiotic resistance. Several groups have performed such studies on Pc promoter activity in different moments and using different approaches [2,3,26,28,29,38,42,43], what limited the comparison of the results. Jové and Colleagues compiled all Pc variants described until that occasion, including those for which transcription activity was unknown and new variants identified in that study, and determined their transcription efficiencies under the same conditions [26], as detailed in Table 2. This study demonstrated that the relative transcription strengths of known Pc variants corroborate those obtained in previous studies [2,3,28], in which PcS was the strongest promoter, followed by PcH2, PcW + P2, PcH1, and PcW. Considering the newly identified Pc variants, PcIn116 presented the lowest transcription efficiency, while PcPUO and PcIn42 strengths were similar to that of PcW [26]. Although PcSS had been previously described as a promoter stronger than PcS [38], Jove and Colleagues demonstrated that its transcription was about 12-fold less efficient than that driven by PcS. The TGN-10 motif provided a significant increase in PcW efficiency, approaching that of PcH2 and two-fold higher than that of PcW + P2, while no relevant effect on the activity of the strongest PcS and PcH2 was observed in the presence of the TGN-10 motif. Conversely, the TAN-10 motif in the PcW_TAN-10_ configuration, severely reduced the PcW activity, while the TTN-10 motif found in the PcH1_TTN-10_ version, slightly increased promoter efficiency similar to that found for PcH1 + P2 (Table 2) [26]. The P2 promoter contributed to a remarkable increase in transcription when associated with PcW and PcH1 versions, however, when combined with the strongest PcS and PcW_TGN-10_ variants, their efficiencies suffered a slight decrease. The newly mutated forms of P2, P2m1, and P2m2 seemed to be inactive [26], however, the P2m3 promoter, found in association with PcW, was able to double promoter strength, approaching the PcW_TGN-10_ expression level (Table 2) [29]. A recent study demonstrated the role of PcH1, PcS, and PcW_TGN-10_ on the expression of a variety of resistance gene cassette arrays in class 1 integron-positive *Proteus* strains [44]. All of them were able to drive cassette expression, and strains containing stronger promoters, such as PcS and PcW_TGN-10_, presented higher antibiotic resistance rates than strains with weaker promoters (PcH1).

In summary, PcS ≈ PcS_TGN-10_ ≈ PcS + P2 > PcH2_TGN-10_ ≈ PcH2 ≈ PcW_TGN-10_ ≈ PcW + P2m3 ≈ PcW_TGN-10_ + P2 > PcH1 + P2 ≈ PcH1_TTN-10_ ≈ PcW + P2 > PcH1 > PcSS > PcPUO ≈ PcW ≈ PcIn42 > PcIn116, and such relative transcription strengths are proportionally reflected on enzymatic activity and antibiotic resistance phenotypes. Therefore, the multiplicity of these Pc sequences with different activities demonstrates that the expression of an antibiotic resistance gene cassette could vary in response to which Pc configuration is found in the integron [26].

The cassette promoters of other RIs, such as classes 2, and 3 integrons, and of the chromosomal integrons of some species have also been characterized, although in a lesser extension relative to class 1 integrons, as discussed below.

#### 2.1.2. Pc Promoter in Class 2 Integrons

The class 2 integron Pc promoter sequence was precisely determined and functionally characterized in studies that used different approaches. These platforms are associated with transposons related to Tn*7* and usually carry *dfrA1*, *sat2*, and *aadA1* resistance gene cassettes followed by the pseudo-cassette *ybeA* of unknown function [45]. Variations in this gene cassette array are rarely reported due to truncation of the *intI2* gene by a premature ochre stop codon (TAA) at position 179, which yielded an inactive shorter protein [46].

Previous studies had pointed to the presence of four potential class 2 Pc promoters (Pc2A to Pc2D) located within the *attI2* region [46,47,48]. However, the configuration and activity of any of these Pc2 have not been assessed in these studies. Subsequently, a fifth putative Pc2 promoter, Pc2E, was found embedded in the *intI2* coding sequence displaying a TGN-10 motif [48] (Table 3).

**Figure 2:**
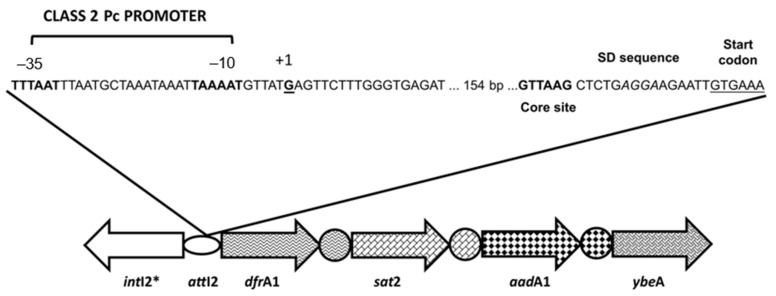
Schematic representation of the class 2 integron and its Pc promoter. The Pc2A promoter sequence and the TSS (+1) are featured. The core site and the ribosomal binding site of the first cassette (*dfrA1*) are highlighted in bold and italics, respectively, while the *dfrA1* start codon is underlined.

Fonseca and Colleagues [49] determined the exact sequence and the activity of a Pc2 promoter by performing in silico promoter prediction analysis and in vitro characterization of the transcription start site (TSS) of the gene cassette array in class 2 integrons (Figure 2). It was demonstrated that this Pc, which corresponded to the previously mapped Pc2A, was responsible for the production of gene cassette transcripts and antibiotic resistance manifestation. This Pc2A (Table 3) resembled σ^70^-dependent promoters, preserving 8 of 12 nucleotides of the consensus sequence –35 TTGACA (16–18 bp) –10 TATAAT. Different from class 1 Pc promoters, which are found embedded in the *intI1* gene [3], the Pc2A was embedded in the *attI2* site, 192 bp upstream *dfrA1*, the first gene cassette usually found in class 2 integrons [45,46]. These findings corroborated previous studies that suggested the existence of a possible promoter region in that recombination site [46,47]. Moreover, the Pc2A was found immediately upstream (five nucleotides) from the characterized TSS of gene cassette array and taking into account that the distance between a promoter and the TSS is usually 7–10 nucleotides, this Pc was assigned to be responsible for transcription of the resistance gene cassettes (Figure 2) [49]. Considering the sequence of the four major class 1integron Pc promoter variants (PcS, PcW, PcH1, and PcH2), the Pc2A was more similar to PcS, presenting three and one polymorphisms relative to –35 and –10 hexamers, respectively.

**Figure 3:**
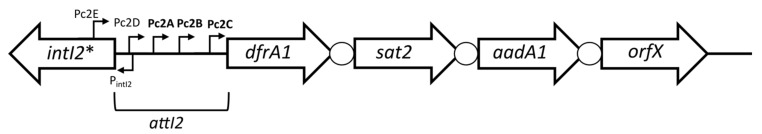
The five Pc2 promoters of class 2 integrons. The truncated intI2* and gene cassettes generally found in class 2 integrons are represented by arrows. The *attI2* and *attC* recombination sites are indicated by a black line and circles, respectively. The four Pc2 promoters (PcA, PcB, PcC, PcD) found in *attI2* region and the Pc2E embedded in intI2* are indicated by broken arrows. The functional promoters (Pc2A, Pc2B, and Pc2C) are highlighted in bold.

Another study addressing this issue [48] experimentally determined the functionality and the relative transcription strength of the five Pc2 promoters, Pc2A to Pc2E (Figure 3). It was demonstrated that the Pc2A, already characterized in Fonseca et al. [49], Pc2B and Pc2C were functional and that the formers have an equal contribution to gene cassette expression, while Pc2C presented a negligible transcription activity (Table 3). This simultaneous occurrence of different active Pc2 promoters is a unique feature of class 2 integrons among RIs/MIs, which could be involved to the differential activation of Pc2A and/or Pc2B under particular environmental conditions [48].

The in silico analysis of hundreds of class 2 integron sequences revealed, in rare cases, the occurrence of polymorphisms in Pc2A and Pc2B promoters, indicating the presence of class 2 Pc variants that could influence promoter strength and antibiotic resistance, as previously noticed for class 1 Pc promoters [26]. These Pc2 variants presented an A/G mutation at the Pc2A –10 hexamer (TAAAGT), and a G/A mutation at Pc2B –35 hexamer (TTATAT), and these variants were named Pc2A-V2 and Pc2B-V2, respectively (Table 3). These rare Pc2 variants were less efficient in transcribing inserted gene cassettes than the canonical Pc2A and Pc2B, and they occurred only when the intact and active *intI2* gene, without the ochre premature stop codon, was present [48,50,51,52,53]. This finding indicated that the existence of these Pc2 variants displaying weaker activity with a consequent decrease of gene cassette expression may be potentially associated with fitness cost reduction.

#### 2.1.3. Pc Promoter in Class 3 Integrons

The class 3 integron was the second RI/MI to have its Pc promoter characterized. The class 3 integron identified in a clinical *Serratia marcescens* strain [54] was submitted to functional characterization to determine IntI3 activity and gene cassette array expression [55]. It was demonstrated that the expression of class 3 integron-containing gene cassettes was controlled by a common promoter, Pc3, which was located just inside the *intI3* gene at a position equivalent to that found for Pc in class 1 integrons. The Pc3 (–35 TAGACA (17 bp) –10 TAGGCT) presented one and three polymorphisms in the –35 and –10 hexamers, respectively, relative to σ^70^ consensus promoter from *E. coli* [3]. The Pc3 strength was experimentally determined by measuring the streptomycin resistance conferred by the *addA2* placed in the first position of the class 3 integron in a controlled system [55], and the results indicated that its strength was equivalent to that determined for PcW + P2 in class 1 integrons [2]. Another study concomitantly revealed the occurrence of a Pc3 variant in a class 3 integron recovered from a *Klebsiella pneumoniae* strain [56], which harbored a gene cassette array distinct from that described in the first reported class 3 integron [54]. This Pc3 variant (–35 TAGACA (17bp) –10 TAGGAT) differed from the canonical Pc3 by a C to A transversion in the –10 region, which might contribute to a differential transcription and, consequently, to variations in the antibiotic resistance phenotype.

#### 2.1.4. Pc Promoter in Chromosomal Integrons

The cassette promoter of CIs has been characterized in some species, being that of *V. cholerae* IntVchA CI studied in more detail [22,57]. The Pc sequence of IntVchA (–35 CTGATT (16 bp) –10 TAGTAT) was determined, and it was shown that this promoter was responsible for transcription of the first cassettes in this CI. Several in vitro approaches demonstrated that the mRNA corresponding to the first cassette in IntVchA started in the vicinity of this Pc promoter, which is located 65 bp upstream of the *attIA* recombination crossover point [22], as found for class 2 integrons [48,49]. Moreover, the genomic analysis revealed that the *attI* and Pc sequences were shared between *V. cholerae* and *V. mimicus* CIs [22], suggesting that both species present similar transcriptional mechanisms.

Although active in several distinct environmental conditions and growth phases, the IntVchA Pc presented maximal levels of induction at the end of the exponential phase in rich media, and under higher temperatures (42 °C) and salinity, which mimicked the environmental conditions faced by *V. cholerae* within the human host. After the intestine invasion, *V. cholerae* produces the cholera toxin that ultimately leads to the increase of salinity concentrations, coinciding with the highest expression of Pc proximal cassettes, which allows the bacteria to adapt to the challenger environment where they will be released. Moreover, it was demonstrated that this Pc promoter was activated by the global regulator cAMP-CRP complex. It directly binds in a CRP box located in *attIA* 41 bp upstream from cassette array transcription start site, overlapping Pc –35 region. The cAMP-CRP regulator complex also has an important role in the *V. cholerae* survival, indicating that the expression of gene cassettes in CIs is tightly linked to conditions requiring adaptation, increasing the chances of bacterial survival in new challenger environments [22].

Different from the Pc promoter of the IntVchA integron, the cassette promoter found in the CI of the environmental *Pseudomonas stutzeri* species (IntIPstQ) was located within its integrase gene. This Pc promoter (–35 TTGAGC (16 bp) –10 TCTGAT) was found 25 bp downstream the *intI* coding gene and it showed to be active in driving transcription of a resistance gene cassette [57].

### 2.2. The Role of Gene Cassette Position Effect on Transcription

There is a clear effect of the cassette position in an integron array on transcription since promoterless cassettes closer to Pc are more efficiently transcribed than distal ones. There is considerable information concerning the position effect on gene cassette transcription in class 1 integrons, but such observation was also made for class 2 integrons [49].

Collis and Hall [2] have examined how the position of resistance gene cassettes inserted in class 1 integrons affected transcription and antibiotic resistance manifestation. They verified that when several cassettes were inserted in the integron, the resistance level increased when the gene was closer to Pc and that such levels reduced in detrimental of the presence of preceding cassettes. Based on these findings, the authors had suggested that not only the relative proximity to Pc influenced gene expression but also that cassettes could decrease transcription of downstream genes placed in distal positions (second, third, and so on) in the array. In that study, Northern blot assays revealed a diversity of transcripts with different lengths, several of them terminating at or near the *attC* site placed on the 3′ end of individual cassettes. Indeed, in spite of their sequence heterogeneity, *attC* sites are characterized by imperfect inverted repeats with a strikingly conserved palindromic organization [6], which may potentially produce stem-loop cruciform structures when in a single strand form, as found during transcription. Therefore, they hypothesized that such hairpin structures could act as Rho-independent transcription terminators in polycistronic transcripts, such as those originating from integron-associated gene cassette arrays, accounting for a decrease in transcription levels of downstream cassettes. However, in spite of this evidence, the role of *attC* in transcription termination was questionable. First, the Rho-independent transcription terminators present stem-loop structures followed by a T-rich region [58], which was not the case for *attC* sites since they are not exactly T-rich sequences. Second, Collis and Hall [2] had observed by Northern Blot that the amounts of mRNA containing only the first cassette or spanning the entire gene cassette array (full-length transcripts) were similar. Third, a more recent study experimentally proved that the transcription of the gene cassettes was not affected by the upstream *attC* sites, independent of their size and sequence composition, since destabilization of different hairpin structures had no impact on the total quantity of transcripts [59]. In fact, a previous study had also demonstrated that the formation of *attC* secondary structures in the mRNA upstream of the *cat* gene had no influence on its transcription [60]. This evidence altogether claims that *attC* sites do not act as transcription terminators of the following gene cassettes in an integron array.

### 2.3. The Role of Cassette-Borne Promoter on Gene Cassette Transcription

**Figure 4:**
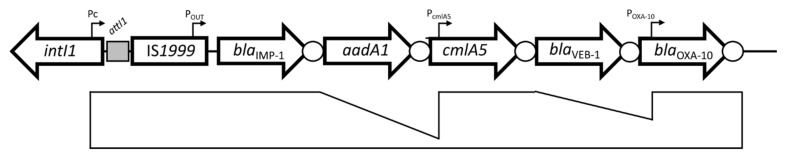
Schematic representation of a class 1 integron with additional gene cassette promoter regions. The Pc, P_OUT_ (supplied at the 3′ end of the insertion sequence), and the cassette-borne promoters P_cmlA5_ and P_OXA-10_ are represented by broken arrows. The influence of cassette-borne promoters in transcription efficiency is represented by black lines.

As discussed above, the transcription of gene cassettes placed in distal positions in an integron array decreases in response to their physical distance relative to the Pc promoter. However, in spite of the general promoterless nature of gene cassettes, some of these structures may harbor longer 5′ untranslated regions (UTRs) that afford the presence of their own specific promoters [47]. The presence of such cassette-borne promoters breaks down the dogma of the cassette position effect, ensuring constitutive expression regardless of Pc configuration and gene cassette position within the array (Figure 4). So far, internal cassette promoters have been described in a diversity of gene cassettes involved with both resistance and non-resistance functions, as addressed below.

The *cmlA1* was the first identified gene cassette containing its own promoter. This promoter (P_cmlA_) showed to be functional as determined by promoter-probe vector assays expressing the *cat* marker gene, and the high chloramphenicol resistance levels. P_cmlA_ was found in the 5′UTR of *cmlA1* cassette, 145 bp upstream its start codon. Afterward, the characterization of other *cmlA* alleles revealed that this gene family was featured by carrying their own promoters. It was demonstrated that the P_cmlA_ was conserved in the *cmlA2* allele, differing by two polymorphisms in the –35 region, while the *cmlA5* allele harbored the canonical P_cmlA_ [41,61,62,63]. In spite of being in the seventh position in the In53 integron array, the *cmlA5* was efficiently transcribed by its active internal promoter (Figure 4) and conferred chloramphenicol resistance, even in distal positions relative to the Pc promoter [41].

The *qac* resistance gene cassettes also carry internal promoters. Both *qacE* and *qacI* present long 5′UTR sequences containing a promoter region that seemed to be active in expressing them, leading to quaternary ammonium compound resistance [2,41,64]. Interestingly, a 101-bp segment (qacE^101^) of the *qacE* 5′UTR has been found fused to the 5′end of several integron-associated gene cassettes, such as *aac(6′)-Ib-cr*, *aacA29a*, *aacA29b*, and *aadA16*, which were transcribed by the *qacE* promoter embedded in this segment [64,65,66,67,68]. Therefore, the qacE^101^ segment consists of a pseudo-cassette acting on transcription of downstream promoterless cassettes, representing an additional strategy to counterbalance the expression level reduction in the integron cassette arrays and/or the weakness of the Pc promoter.

The presence of a cassette-borne promoter has also been identified in the context of class 2 integrons. Biskri and Mazel [47] demonstrated that the *ereA* gene cassette, placed in the second position of a class 2 integron array, harbored a functional promoter in its 5′UTR, placed 21 bp upstream the coding region.

Most of these cassette-borne promoters reported to date had been characterized by means of in silico analysis and the indirect observation of antibiotic resistance phenotype in controlled systems [41,47,62,63]. Fonseca et al. [69] performed in silico, several in vitro and in vivo analyses to fully characterize an internal promoter found in the atypical *qnrVC1* gene cassette, involved with decreased susceptibility to quinolones [10]. The *qnrVC1* was the second cassette in a class 1 integron array, preceded by *aadA2*. The in vitro assays to assess their relative transcription revealed that both gene cassettes produced similar transcript amounts, indicating the presence of an internal promoter in *qnrVC1*. This gene cassette presented an unusually long 5′ UTR of 216 bp, and in silico approaches revealed the presence of a potential promoter in this region placed 29 bp upstream *qnrVC1* start codon. This promoter (P*_qnrVC_*) was related to *E. coli* σ^70^-dependent promoters, preserving 8 of the 12 canonical nucleotides of the –35 and –10 hexamers. The determination of *qnrVC1* TSS by 5′RACE revealed that P*_qnrVC_* was 14 bp upstream the +1 position of the transcript, confirming that this promoter was responsible for driving *qnrVC1* transcription. Moreover, it was demonstrated that P*_qnrVC_* was able to promote the expression of the *cat* gene in promoterless-probe vectors, conferring chloramphenicol resistance in vitro. These findings, together with the production in vivo of fluorescence by *E. coli* cells due to the transcriptional fusion of P*_qnrVC_* with the *gfp* reporter gene, proved that P*_qnrVC_* was functionally active and was responsible for *qnrVC1* expression and quinolone resistance manifestation [69]. It was also demonstrated that P*_qnrVC_* versions were distributed among other *qnrVC* alleles [10,11,69,70,71], ensuring their expression and emergence of resistance phenotypes in distinct genetic contexts and species.

In the context of CIs, cassettes placed in very distal positions relative to Pc, which would be silenced, might be efficiently expressed by the presence of internal promoters. In fact, functional studies of large CIs from different species have demonstrated that a great proportion of gene cassettes, mainly those coding for toxin-antitoxin (TA) systems, were efficiently transcribed due to the presence of cassette-borne promoters, such as *brnT-brnA*, *vapC-abrB/mazE/spoVT*, and *relE-parE* [4,72,73,74,75]. TA systems avoid the entrance of mobile genetic elements by horizontal gene transfer and contribute to the genome stabilization [72,73,74]. For example, the TA systems encoded by *phd-doc* and the *ccd_Vfi_* cassettes found in *V. cholerae* and *V. fischeri* CIs were efficiently expressed by their own active promoters. Similarly, another study demonstrated that the TA loci *relBE1* and *parDE1* cassettes carried in the *V. vulnificus* CI were expressed in vivo and these active TA systems counterbalanced the extent of deletions catalyzed by the integrase [73]. The constitutive expression of such TA cassettes observed in different *Vibrio* species indicated that these systems have a relevant role in the stabilization of CI-associated gene cassettes by minimizing large-scale cassette loss without hindering the microevolution of these genetic platforms mediated by integrase activity [73,74].

Moreover, additionally to TA gene cassettes, other studies experimentally demonstrated that most of the gene cassettes in large CI arrays were transcribed by their own promoters. For example, it was verified that most of the 116-gene cassettes in the CI array of *V. rotiferianus* DAT722 were widely transcribed due to the presence of numerous and diverse internal promoters across the array and that these cassettes were differentially transcribed in response to promoter strengths and to variations in environmental conditions such as thermal and oxidative stress. Therefore, these bacteria harboring CIs have the potential to benefit from the concomitant manifestation of multiple functions irrespective of cassette location within the array. Consequently, the presence of larger functional cassette arrays can provide an increased repertoire of adaptive capabilities, conferring complex phenotypes and multiple selective advantages to the host organism, which could explain the prevalence of CIs in the environment [76].

Therefore, the occurrence of internal cassette promoters is an extra element in the evolution of the adaptive phenotypes, since they guarantee the transcription even of genes that are distal relative to Pc, minimizing the position effect.

### 2.4. Transcription of Gene Cassettes Independent of Pc and Cassette-Borne Promoters

#### 2.4.1. Insertion Sequences Elements

Promoter sequences other than Pc and cassette-borne promoters could also have a relevant role in the expression of promoterless gene cassettes, which also breaks the principle of the cassette position effect. Such promoters are most commonly supplied by the insertion of mobile genetic elements. Several insertion sequences (ISs), such as IS*1999*, IS*Ecp1*, IS*Kpn23* carry complete and functional outward-oriented promoters (P_OUT_) that enhance the expression of downstream antibiotic resistance genes and ensure the emergence of resistance phenotype (Figure 4) [77,78]. Otherwise, some ISs, such as IS*1*, IS*257*, and IS*Aba125* supply the –35 hexamer, generating a hybrid active promoter when adjacent to a downstream –10 element with proper spacing [79,80,81]. It was the case of the aminoglycoside resistance *aphA-6* gene, which was expressed from a promoter composed by a –35 hexamer located in the 3′ end IS*Aba125* and a –10 hexamer in the *aphA-6* flanking sequence [81].

The transposable elements called Insertion Sequence Common Regions (IS*CR*s) belong to the IS*91*-like family elements that act by a mechanism of rolling-circle transposition. The IS*CR*s code for a putative transposase followed by a recombination crossover site (RCS), and they have been involved with the acquisition and mobilization of antibiotic resistance genes [82,83,84]. Among the IS*CR*s, the IS*CR1* is always associated with atypical class 1 integrons, named complex integrons, which present partial duplications of the 3′CS, separated by IS*CR1* associated with antibiotic resistance genes. Several studies experimentally verified that IS*CR1* contained two active outward-oriented promoters (P_CR1-1_ and P_CR1-2_) contributing to the expression of numerous unrelated antibiotic resistance genes found in complex class 1 integrons from different bacterial species, such as the *qnrA*, *dfrA10*, *dfrA19*, *bla*_CTX-M-2_, and *bla*_CTX-M-9_ [85,86,87,88]. However, in spite of the role of IS*CR1* on the expression and manifestation of antibiotic resistance, it had been verified that the relative transcriptional strengths of P_CR1-1_ and P_CR1-2_ were considerably lower than those observed for other IS elements. A previous study verified that the promoters of IS*Aba1*, IS*Ecp1*, and IS*Aba125* presented similar strengths, which was intermediate between those of the variants PcS and PcW_TGN-10_ found in class 1 integrons, but were 30-fold stronger than those from IS*CR1* [89].

#### 2.4.2. Group II Intron Elements

**Figure 5:**
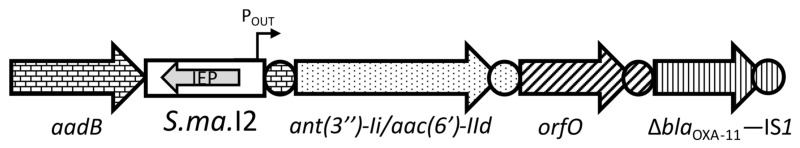
Schematic representation of a class 1 integron carrying the Group IIC-*attC* intron. The *S.ma*.I2 insertion that split the *aadB* gene from its cognate *attC* site and the presence of P_OUT_ promoter provided by this Group IIC-*attC* intron involved with the expression of downstream cassettes are indicated.

Another example of mobile genetic elements contributing to promoterless gene cassette expression is Group II intron elements [90]. Group II introns are mobile genetic elements pointed as the ancestors of spliceosomal introns and retrotransposons in eukaryotes [91]. Among several Group II intron types, the Group IIC introns are featured by invading intergenic regions usually after inverted repeat sequences, such as *attC* sites, and in this case, they are named group IIC-*attC* introns [92]. The *S.ma.*I2 Group IIC-*attC* intron was found splitting the first resistance gene from its cognate *attC* site in an integron of *S. marcescens* [90]. Léon et al. [93], revealed that this *S.ma*.I2 insertion disrupted the transcription of downstream gene cassettes from the integron Pc promoter (PcW + P2 configuration), probably due to the increase in the physical distance between cassette and Pc. However, in silico and in vitro analyses revealed that *S.ma*.I2 insertion supplied a functional internal outward-oriented promoter (P_out_) that was active in expressing promoterless gene cassettes that had been silenced the insertion of the intron (Figure 5). Moreover, other P_out_ promoter versions sharing sequence similarity with the *E. coli* promoters were conserved among group IIC-*attC* intron sequences. These promoters presented different transcription strengths that ranged between those observed for PcS and PcW from class 1 integrons [93].

#### 2.4.3. ORF-Less Cassettes

**Figure 6:**
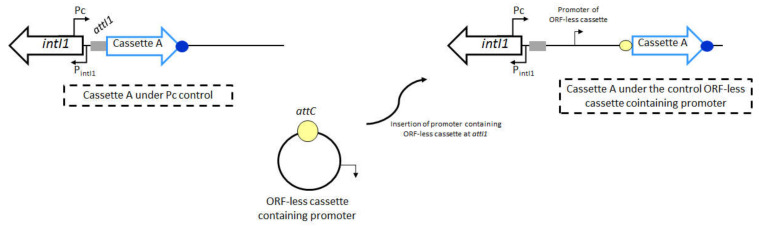
Schematic representation of the acquisition dynamics of a promoter-containing ORF-less cassette by a class 1 integron. The insertion of the promoter-containing ORF-less cassette into *attI1* site disengages the original first gene cassette A (blue arrow) from Pc promoter but provides a new promoter region.

It has been documented the existence of non-coding cassettes in integrons from distinct environmental metagenomes. These atypical cassettes, named ORF-less cassettes, were devoid of open reading frames, but carried classical *attC* site in their 3′ end [94,95]. Moreover, some of them encode regulatory RNAs, such as ORF-less cassettes identified in the *Xanthomonas campestris* pv. *campestris* integron that were involved in the regulation of virulence [96]. A recent study using a dual report plasmid approach identified functional promoters in ORF-less cassettes belonging to *Treponema* species from the oral metagenome. This study also demonstrated that these promoters resembled σ^70^ *E. coli* consensus sequences, and they, indeed, were functional in that organism. Moreover, these ORF-less cassettes were characterized by providing promoter sequences at the antisense strand. Therefore, once in the first position of the integron array, these ORF-less cassettes could potentially increase expression of the integrase with a consequent increase of recombination events, leading to the acquisition of new gene cassettes at the first position. When found embedded in cassette array, these ORF-less cassettes could act on transcription of distal gene cassettes relative to Pc (Figure 6). Therefore, these ORF-less cassettes might have an important role in gene expression regulation, allowing bacteria to adapt to multiple stresses within complex physicochemical environments, such as that of the human oral cavity [75].

#### 2.4.4. Fused Gene Cassettes

Gene cassettes generally include a single gene and an *attC* site. However, cassette fusions may arise in several ways, such as slippage during DNA replication caused by *attC* stem-loop structures, or as a result of illegitimate integrase-mediated intermolecular recombination events between internal regions of *attC* and *attI* sites, which lead to *attC* site loss (partial or total) from the upstream cassette [5]. In this case, both complete coding regions are retained in a unique cassette, creating permanent gene arrays comparable to bacterial operons, which remain mobilizable by the *attC* site from the second cassette [5,27].

Several fused cassettes have been documented, and in some cases, the fused genes could be expressed by the presence of upstream promoter regions. It has been shown that the fused gene cassette *bla*_OXA-10_/*aadA1*, created by the deletion of *bla*_OXA-10_ *attC* site, was transcribed by their own promoter sequence present upstream *bla*_OXA-10_ cassette [41]. In Tn*1331*, the promoterless *bla*_OXA-9_ gene, found immediately downstream of an *aadA1* cassette with a truncated *attC* site, was transcribed from both the Pc and from an additional promoter located at the 3′end of the *aadA1* coding region [97]. Céntron et al. [90] demonstrated that the *ant*(*3*)*-Ii/aac*(*6*)*-IId* fused cassette was transcribed by a promoter provided by an upstream Group II intron, generating a bifunctional protein that retained both ANT(3)-I and AAC(6)-II activities, as demonstrated by the observed extended aminoglycoside resistance phenotype.

**Figure 7:**
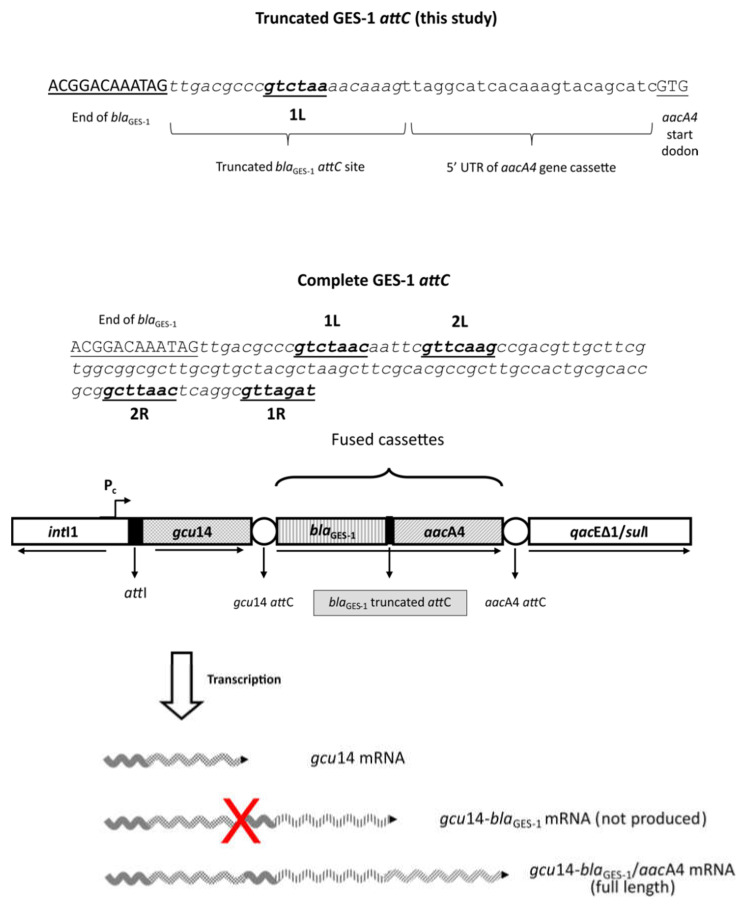
The *bla*_GES-1_/*aacA4* fused cassette formation and their transcription dynamics. Truncation of GES-1 *attC* site involved with *bla*_GES-1_/*aacA4* fused cassette formation. Sequences of the complete and truncated GES-1 *attC* sites are shown. The *bla*_GES-1_ and *aacA4* partial coding regions are underlined and in uppercase. The *attC* sites are italicized, and their core sites (1L, 2L, 2R, 1R) are underlined and in bold. The transcription dynamics of *gcu14*-*bla*_GES-1_/*aacA4* gene cassette array. Transcription direction is represented by horizontal arrows. The scheme shows that all mRNA molecules originating from Pc promoter corresponded to *gcu14* monocistronic or full-length polycistronic transcripts (curved lines), demonstrating that the fusion *bla*_GES-1_/*aacA4* is always transcribed together (bottom).

However, in spite of the presence of specific promoters in several fused cassettes, studies on the transcription dynamics of fused gene cassettes in integrons were scarce. A previous study measured the relative expression of the *bla*_GES-1_/*aacA4* fused cassette. This fusion was placed in the second position of a class 1 integron, preceded by the *gcu14* cassette of unknown function [98]. This fusion was created by partial loss of 91 bp of GES-1 *attC* site, retaining the entire coding regions of both *bla*_GES-1_ and *aacA4* (Figure 7). This study experimentally revealed that the *gcu14* cassette, found in the first position closer to the PcW_TGN-10_, had an increased transcription relative to *bla*_GES-1_ and *aacA4*. On the other hand, similar transcript amounts were observed for *bla*_GES-1_ and *aacA4*, both alone and in the fused form, indicating that that the fused genes were transcribed together. Northern Blot revealed the production of both monocistronic and full-length polycistronic transcripts of gene cassette array, as found elsewhere [2]. The monocistronic transcripts corresponded in size to the *gcu14* gene cassette, but never to *bla*_GES-1_ or *aacA4* alone. In fact, *bla*_GES-1_ and *aacA4* were only found in full-length polycistronic transcripts corresponding to the entire gene cassette array *gcu14-bla*_GES-1_/*aacA4*, demonstrating that these genes retained in the fusion were always co-transcribed [98]. Moreover, the identification of polycistronic transcripts comprising the *gcu14* cassette, which harbors a complete *attC* site, gave support to the previous evidence that *attC* does not act as a transcriptional terminator [59].

### 2.5. Regulation of Integrase Transcription in MI and CI by Stress Response Mechanisms

#### 2.5.1. The Role of SOS Response

The SOS response is a complex regulatory network involved with DNA repair in response to damage or bypassing lesions, directly linked with adaptation and emergence of phenotypes clinically relevant [17,18,19,99]. The LexA is the main transcriptional repressor that controls the SOS response, and the derepression of this system is associated with the RecA-dependent cleavage of LexA induced by the presence of abnormal rates of single-stranded DNA (ssDNA), which is a signal of genotoxic stress to the cell. The LexA binds specifically to a palindromic region of 16 bp located in the vicinity of the RNA polymerase binding site, blocking gene transcription by interfering with RNA polymerase activity [100].

**Figure 8:**
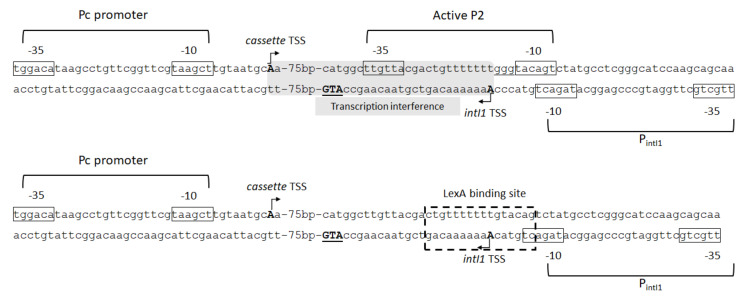
Sequence and organization of the Pc, P2 and P_intI1_ promoters and the LexA binding site in class 1 integron. The –35 and –10 hexamers of all promoters are boxed and the GGG insertion that creates the active P2 is underlined. The transcriptional initiation sites (TSS) are indicated in bold with broken arrows. The integrase start codon is shown in bold underlined uppercase letters. The overlapped region corresponding to transcripts originating from the inversely oriented Pc and P_intI1_ that leads to transcription interference is shaded in grey (**top**). The sequence and organization of the LexA binding site are highlighted by a dotted box (**bottom**).

Curiously, a conserved LexA-binding motif was identified overlapping the –10 hexamer of the integrase promoter region (P_int_) in both CIs and MIs (Figure 8) of clinical and environmental sources, and some studies experimentally determined that the LexA specifically binds to this motif in a large fraction of integrases [17,19]. In fact, phylogenetic analysis indicated that the LexA regulation of *intI* genes is ancient and that it is a widespread and conserved phenomenon among integrons [19].

**Figure 9:**
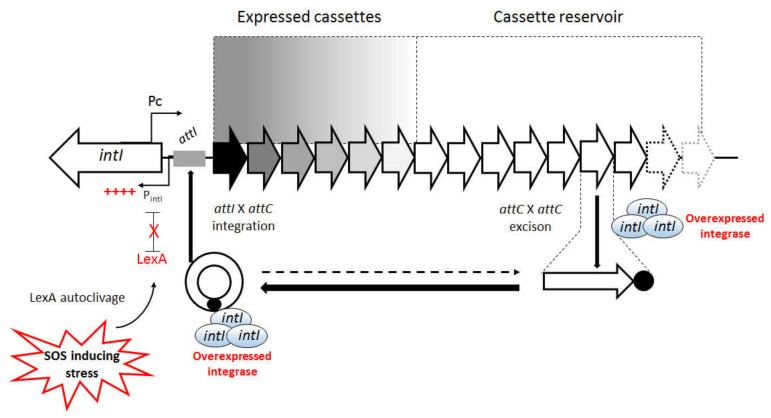
Schematic representation of the interplay between the SOS response induction and the integron activity. The basic structure of integrons (integrase *intI* gene, the *attI* recombination site, and the Pc and P_intI_ promoters) is indicated. The first cassettes expressed by the Pc promoter are indicated by arrows filled from faded black to light gray colors representing the most and least expressed cassettes, respectively, while distal silenced cassettes are represented by blank arrows. The mechanism of integrase expression regulation by SOS response is shown. SOS stress response inducers lead to LexA autoclivage, releasing the P_intI_ promoter and derepressing integrase expression. Circular cassette intermediate resulted from excision (*attC* X *attC* recombination) of distal positions and its possible reinsertion (*attI* X *attC* recombination) next to Pc mediated by integrase activity upon derepression by SOS induction are demonstrated.

It was demonstrated that in normal conditions, the LexA remained bound to its motif, avoiding the recruitment of RNA polymerase to P_int_ and, therefore, repressing integrase transcription. However, upon induction of SOS response, the cleavage of LexA released the P_int_ promoter, allowing integrase transcription. The in vitro assays revealed that this derepression by SOS induction resulted in a significant increase of excision rates by integrase (Figure 9).

As aforementioned, the newly acquired cassettes in an integron are more efficiently expressed by the Pc promoter, which can become silenced by continuous integrase recombination events that move them to more distal positions in the array [2,7]. Under steady-state conditions, SOS repression of *intI* maintains the architecture of the existing cassette arrangement. However, the induction of SOS response by stressful conditions, such as DNA damage by the antibiotics ciprofloxacin, trimethoprim, β-lactams, and metronidazole, leads to antibiotic resistance emergence [100,101,102,103,104]. The induced SOS system upregulates the integrase expression when innovations and adaptation to novel situations are needed, ensuring cassette shuffling, which allows expression of previously silenced genes, and acquisition of new ones from the surrounding bacterial communities (Figure 9) [17]. Furthermore, the gene cassettes that have been temporarily silenced in distal positions may also contribute to bacterial evolution, since they represent a valuable source of variability, accounting for the spread of strains carrying cryptic resistance determinants [19,104]. In fact, a previous study demonstrated that the antibiotic-induced SOS response led to the one-step emergence of the ceftazidime-resistant phenotype during the course of human infection, due to the reordering of a previously silenced gene cassette by an active class 1 integrase. They observed that the previous treatment of a patient with metronidazole and ceftazidime induced the SOS response, with the concomitant activation of integrase expression. This integrase excised an upstream cassette allowing the proper and full expression of the *bla*_OXA-28_ gene, resulting in the manifestation of the observed ceftazidime resistance. Therefore, this study showed that metronidazole induction of the SOS response favored the emergence of highly resistant bacteria within a patient that could be disseminated under selective antibiotic pressure [104].

Since the SOS system is activated by the accumulation of ssDNA molecules, horizontal gene transfer events that lead to the transient formation of the exogenous ssDNA could also contribute to the induction of SOS response. In fact, in vitro, and in vivo studies revealed that the SOS response was induced in *E. coli* and *V. cholerae* cells during plasmid conjugative transfer leading to the upregulation of *intI1* and *intIA* expression, which, in turn, triggered cassette recombination in both MI and CI platforms [18]. Besides conjugation, ssDNA uptake by naturally competent cells also induces SOS system and integrase expression, demonstrating the role of transformation events in the regulation of integron functionality [21].

This intricate regulatory mechanism is also demonstrated by the significant correlation observed between the loss of LexA repression and the integrase inactivation, indicating that recombination events mediated by a constitutively expressed integrase may be deleterious to the host [19]. However, this lack of LexA regulation can be accepted or selected upon providing adaptive advantages. For example, it had already been demonstrated that the creation of a functional P2 promoter, by the insertion of a GGG triplet in its shortened spacer region, evoked heightened rates of resistance gene cassette expression in class 1 integrons harboring the weak PcW configuration [2,3]. However, the GGG insertion disrupts the LexA binding site, preventing LexA from binding to P_int_, which abolishes integrase regulation (Figure 8). Therefore, this phenomenon suggested that LexA repression may be abolished under strong selective pressures to promote variability and increased basal transcription levels of antibiotic resistance genes. In this case, the maintenance of integrase activity providing cassette shuffling and phenotypic innovation must be preferable to the loss of integron functionality under changing selective environments, such as the clinics [19]. Despite these specific cases, there is a clear selective pressure regarding the conservation of the LexA-dependent integrase regulation. For example, in the CIs, this pressure is represented by the need in stabilizing these large platforms in the genome [72]. In MIs, this selective pressure might favor inactivated integrons that are able to run into recombination activity when adaptive innovation is needed [19]. It means that the SOS-dependent regulation of integrase and antibiotic resistance gene expression leads to gene silencing at no biological cost until activation by selective antibiotic pressure, while other adaptive traits continue to be expressed [17,105].

Another example of the link between the SOS response and the evolution of antibiotic resistance is the association of this system with the expression of *qnrB* genes. The *qnrB* alleles are frequently found associated with IS*CR1* in complex class 1 integrons and confer resistance to quinolones, such as ciprofloxacin, by protecting the antibiotic targets. An internal promoter region is conserved in all *qnrB* alleles as well as a sequence homologous to the gammaproteobacteria consensus LexA-protein-binding site found in the vicinity of these *qnrB* promoters. In this case, as found for *intI* genes, the *qnrB* expression is negatively regulated by the activity repression of LexA [105]. Interestingly, the SOS system is known to be induced by ciprofloxacin [102], which upregulates the expression of its own resistance determinant (*qnrB*).

#### 2.5.2. The Role CRP-cAMP Catabolite Repression Pathway and Environmental Physicochemical Conditions

**Figure 10:**
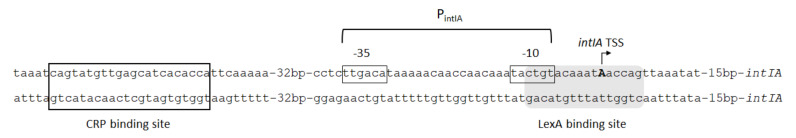
Sequence and organization of the P_intIA_ promoter and the LexA and cAMP-CRP binding sites in the *attIA* of the *V. cholerae* chromosomal integron. The CRP and the LexA binding sites are boxed and shaded in grey, respectively. The *intIA* transcriptional start site (TSS) is featured in bold and a broken arrow. The P_intIA_ –10 and –35 hexamers are highlighted.

As aforementioned, the ssDNA uptake by transformation is another inducer of the SOS response and integron functionality activation [21]. Regulation of natural competence depends on several factors, one of them is the regulon of the receptor protein (CRP) of cyclic AMP (cAMP), involved with carbon catabolite repression [106]. Reduced levels of carbon sources in the environment trigger a regulatory cascade that culminates in the increase of intracellular cAMP. Such heightened levels of cAMP ultimately modulate transcription of several genes associated with physiologic features and environmental stimuli, such as adaptation to starvation, quorum sensing, virulence, and competence [21]. Therefore, as found for the SOS system, the cAMP-CRP regulon can also be considered a stress response mechanism. A previous study observed that the expression of *intIA* from *V. cholerae* CI varied in detrimental of carbon source, indicating that the integrase expression is regulated by cAMP-CRP. Indeed, Baharoglu et al. [21] demonstrated the existence of a 22 bp-cAMP-CRP binding site conserved in the vicinity of *intIA* promoter (44 bp and 69 bp upstream P_intA_ and LexA binding site, respectively) (Figure 10), and they observed the effective binding of the cAMP-CRP complex to this region, impacting on integrase expression. Afterward, it has been verified that Pc from *V. cholerae* CI is also activated by the cAMP-CRP complex, which binds to this same CRP box and upregulates gene cassette expression (Discussed above in topic 2.1.4) [22]. However, the absence of a CRP binding site in the *attI1* site encompassing the *intI1* promoter suggests that class 1 integrons are not under cAMP-CRP regulation [107]. Therefore, besides SOS response activation, integrase expression in *V. cholerae* CI was also submitted to cAMP-CRP regulation (Figure 10).

Moreover, as found for Pc promoters in *V. cholerae* CI, the P_intA_ is also highly induced under physicochemical conditions that mimic the human intestine, such as high temperature and salinity. In this way, an increase in the rates of acquisition/reordering and expression of gene cassettes is observed in response to the concomitant induction of P_intA_ and Pc promoters, allowing *V. cholerae* to rapidly adapt to the challenges of its next environment after being excreted from the human host [22].

#### 2.5.3. The Role of the Stringent Response

Biofilms are represented by microbial communities typically encased in an auto-produced extracellular matrix, which creates micro niches that facilitate the occurrence of horizontal gene transfer, contributing to the spread of antibiotic resistance [108]. They also present an increased level of expression of stress-related genes compared to those observed in the planktonic counterparts. In fact, in the environment represented by biofilms, some bacteria may undergo nutrient starvation that stimulates a stringent response [109], resulting in a complex regulatory cascade that increases the concentration of guanosine pentaphosphate or tetraphosphate ((p)ppGpp) and polyphosphate (polyP). The Lon protease, a global regulator of the stress response, binds to the accumulated polyP, forming the poly-P–Lon complex, primarily involved with the degradation of free ribosomal proteins [110]. The (p)ppGpp is an alarmone that causes the inhibition of RNA synthesis during starvation [111], and upregulates several genes involved in stress response, such as the SOS system, leading to *intI1* upregulation by poly-P–Lon complex [107]. Therefore, the stringent response, commonly occurring in biofilms, is another regulatory pathway that increases integrase expression by both SOS induction and by a biofilm-specific mechanism involving the Lon protease. In this way, biofilms favor gene cassette acquisition/rearrangement, resulting in the emergence of antibiotic resistance and other adaptive functions, in bacteria harboring class 1 integrons [107].

#### 2.5.4. The Role of Other Stress Response Regulators

Bacteria frequently face environmental changes to which they reshape by using alternative σ factors, such as RpoS, to differentially express their genes. RpoS is a central regulator of the general stress response, and one of the major transcriptional regulators of the genes required for bacterial adaptation. Transcription of *rpoS* in *Pseudomonas* spp. is regulated by PsrA, and a recent study demonstrated that RpoS and PsrA were involved with the upregulation of integrase transcription in this genus. Since the *intI1* gene lacks PsrA and RpoS binding motifs [112], it has been proposed that RpoS and PsrA regulators indirectly regulated *intI1* transcription. In fact, Novovic et al. [113] verified that in conditions of stress response during the stationary phase, PsrA and RpoS indirectly stimulated *intI1* transcription through regulation of *lexA* expression in association with additional regulators. It has been demonstrated that *psrA* and *lexA* genes share the same intergenic region that is targeted by PsrA. In this way, PsrA blocks its own promoter by preventing the binding of the RNA polymerase. However, at the same time, the binding of PsrA leads to a conformational structure modification that facilitates the RNA polymerase binding to the opposite chain, inducing *lexA* transcription. In this way, PsrA upregulates the transcription of the *lexA* gene while simultaneously repressing its own expression, ultimately leading to integrase transcription [113]. Additionally, Cagle et al. [20] identified binding sites for the nucleoid proteins H-NS, IHF, and FIS in and near the integrase (P_intI1_) and cassette promoters (Pc/Pc + P2), which differentially regulated *intI1* expression in class 1 integrons. This regulatory mechanism will be discussed in more detail below since it involves the simultaneous regulation of P_intI1_ and Pc promoters.

Finally, here, we compiled overall information from previous studies concerning the complex regulatory network of integrons. It was demonstrated that integrase genes are multiply regulated by different environmental conditions (temperature, salinity, growth phase), by mechanisms of the stress response (SOS system, cAMP-CRP regulon, stringent response, and RpoS), and by global regulators involved with several physiologic and metabolic pathways (FIS, H-NS, and IHF) [17,18,19,20,21,22,99,107,113]. Therefore, these findings altogether clearly demonstrated the elegant and intimate interplay of the external environment and host cell physiology with genetic adaptability and integron dynamics.

### 2.6. Interplay between Integrase and Gene Cassette Expression in Class 1 Integrons

#### 2.6.1. Integrase Activity Is Linked and Inversely Correlated to Gene Cassette Expression

As extensively discussed above, dozens of Pc variants may naturally occur in class 1 integrons due to point mutations in the –35 and –10 hexamers [26]. Since the Pc promoter is found embedded in the 5′ end of the *intI1* coding sequence, the polymorphisms that give rise to the different Pc promoter variants may also lead to non-synonymous mutations in the *intI1* gene, resulting in amino acid changes in integrase. These alterations in the class 1 integrase interfere with its recombination activity and determine different catalytic *intI1* variants with varying efficiencies of excision mediated by *attC* X *attC* recombination. The same IntI1 variant may be defined by different Pc promoters, in which 13 Pc versions determine 10 variants of IntI1. Three of these 10 integrase versions, with amino acid changes occurring at positions 32 and 39 (IntI1R32_H39, IntI1R32_N39, and IntI1P32_H39) represent the great proportion of IntI variants. Jové et al. [26] estimated the ability of these integrase variants in excising gene cassettes. They revealed that the substitution of an arginine (R) by a proline (P) at residue 32 and of histidine (H) by an asparagine (N) at residue 39 led to a remarkable reduction in integrase excision efficiency. Comparisons with the IntIA from *V. cholerae* CI revealed that these amino acid positions were within an α-helix involved in *attC* binding [114], which is also conserved in the predicted structure of IntI1 [115]. Therefore, amino acid changes at positions 32 and 39 probably disturb the *attC* binding by IntI1, resulting in the low excision efficiency observed for these integrase variants. On the other hand, such amino acid changes had a less relevant impact on the efficiency of gene cassette integration mediated by IntI1 variants, indicating that *attI* X *attC* (integration) and *attC* X *attC* (excision) recombination reactions may involve different regions of the integrase [26]. Therefore, an inverse correlation between Pc promoter strength and the integrase excision activity was observed, since the polymorphisms that defined weaker Pc promoters also defined more active integrase enzymes, while those determining the strongest Pc configuration led to integrase variants with lower activity. The IntI1R32_H39, the variant with the most efficient excision activity, correlated with the presence of Pc configurations (PcW and PcH1) involved with weak transcriptional strength. In this case, the low expression level of resistance genes conferred by weak Pc versions might be compensated by the high excision rates evoked by the variant IntI1R32_H39, which confers a remarkable ability of cassette reassortment that may bring previously silenced cassettes closer to Pc, enabling bacteria to adapt more rapidly to harsh environments. On the other hand, in a challenger scenario of heavy antibiotic pressure, stronger Pc configurations, such as PcS and PcW_TGN__–__10_, are selected in order to express antibiotic resistance gene cassettes more efficiently, in detriment of IntI1 excision activity, leading to the maintenance of advantageous cassette arrays. This assumption is in accordance with the fact that integrons carrying the integrase variants IntI1R32_N39 or IntI1P32_H39 generally have arrays with a greater number of gene cassettes than those harboring IntI1R32_H39, since the formers are less efficient in excising gene cassettes [26].

A previous study evaluated the efficiency of rearrangements mediated by different IntI1 variants under antibiotic selective pressure. It was demonstrated that, independently of the integrase variant present, the IntI1 was able to perform multiple cassette excision events to bring a transiently silenced *catB9* gene cassette closer to Pc in response to chloramphenicol presence. However, the occurrence of multiple gene cassette arrangements was observed only when the most active integrase variant IntI1R32_H39 was present [26]. In this case, IntI1 preferentially excised the cassettes immediately preceding *catB9* rather than excising *catB9* and reinserting it closer to Pc, since it is less cost-effective considering that only one recombination event takes place. Additionally, although the dynamics of cassette shuffling were higher when IntI1 was found derepressed or overexpressed, such recombination events under selective pressure were observed even when IntI1 was weakly expressed in response to LexA repression [116]. Interestingly, in all rearrangements observed in that study, the *catB9* was brought to the second cassette array position, downstream of *aac(6′)-Ib*. This could be due to the lower efficiency of the integrase in catalyzing excision of *aac(6′)-Ib* in the first position, which involves *attI* X *attC* recombination [23], or to the length and structure of the folded bottom strand of the *aac(6′)-Ib* and *catB9 attC* sites, which could be poorly recognized by IntI1 [117].

A recent study established another interesting interplay between cassettes expression and integrase activity. As extensively discussed above in the previous topic, the *intI1* expression is tightly regulated in class 1 integrons by LexA repressor [17]. The LexA protein is absent in *Acinetobacter baumannii*, suggesting that *intI1* expression in this species is probably constitutive. However, a recent study demonstrated that class 1 integrons identified in *Acinetobacter* predominantly harbor the PcS configuration, the strongest cassette promoter variant [43]. As already mentioned, the PcS configuration determines the least efficient integrase variant (IntI1R32_N39) [26] and, consequently, the lowest fitness cost for bacteria. Therefore, this finding suggested that the PcS prevalence in *A. baumannii* could be associated with the reduced fitness burden to evade the deleterious activity of integrase. Additionally, this study also verified that despite the strong nature of the PcS promoter, its transcription efficiency was weaker in *A. baumannii* than in *E. coli*. Although *A. baumannii* and *E. coli* RNA polymerases and σ^70^ factors share considerable conservation, such inter-species differences in Pc activity could result from differences in their transcriptional machinery [118]. Therefore, this regulatory mechanism represents an adaptive response designed to reduce the fitness cost and contribute to integron stabilization in this species [43].

#### 2.6.2. Transcription Interference between P_intI1_ and Cassette Promoters in Class 1 Integrons

It is already known that, in the case of class 1 integrons, the Pc promoter is found embedded in the *intI1* gene and that Pc and P_intI1_ drive transcription in opposite orientations [2,3], with the corresponding transcription initiation sites separated by 100 bp. Thus, when both P_int1_ and Pc promoters are active, convergent transcription takes place, resulting in transcriptional interference due to sense–antisense mRNA interactions (Figure 8). In fact, it was verified that P_int1_ is more efficient in driving *intI1* transcription in the absence of an active Pc promoter. A previous study demonstrated that P_intI1_ is susceptible to transcription interference, which preferentially occurs in the presence of the strong PcS. Due to the high transcription levels that PcS evokes, this is the only Pc variant able to influence negatively *intI1* expression even in the absence of LexA repression [101]. These findings indicated that the *intI1* is less transcribed in the presence of strong Pc promoter versions, which is expected in transcription interference events, where the activity of a promoter is negatively influenced by that of a stronger one [119]. However, although the presence of an active P2 resulted in LexA binding site disruption, derepressing *intI1* expression, it does not interfere with P_intI1_ promoter activity, demonstrating that the 3bp-overlapping between P_intI1_ and P2 –10 hexamers was not sufficient to provoke transcriptional interference. On the other hand, P_intI1_ had no impact on gene cassette expression driven by Pc variants or P2, which is in agreement with the fact that Pc is not regulated by SOS response. However, in a class 1 integron carrying PcS, gene cassette and *intI1* transcription is coupled due to the negative interference of PcS on P_intI1_ activity by transcription interference.

Additionally, transcription interference evoked by PcS and P_intI1_ activity proved to be an important mechanism of integron regulation in *A. baumannii*. As mentioned in the previous topic, *A. baumannii* lacks the LexA repressor, allowing constitutive integrase expression in this species. In this case, the transcriptional interference mediated by PcS led to a decrease in integrase expression, counterbalancing the lack of LexA-driven integrase repression. This modulation avoided the deleterious effects of unregulated integrase, explaining the remarkable predominance of PcS in class 1 integrons from *A. baumannii* [43]. Therefore, besides Pc and P2 promoters play a relevant role in gene cassette transcription, they are also linked with the integron functionality [101].

#### 2.6.3. Global Regulators Concomitantly Control the Activity of P_intI1_ and Cassette Promoters in Class 1 Integrons

FIS, IHF, and H-NS are multifunctional proteins that act as recombination accessory elements, nucleoid compaction proteins, and global transcriptional regulators of many distinct metabolic pathways [120]. Cagle et al. [20] demonstrated that these global regulators concomitantly interfere with integrase and cassette expression in class 1 integrons, modulating cellular integrase concentrations in response to different host growth phases. The FIS protein repressed both integrase and cassette expression and seemed to stabilize LexA occupancy via their closely adjacent sites in the integron promoter regions, contributing to maximum LexA repressor activity. Conversely, IHF acted as a LexA-independent weak activator of P_intI1_ and cassette promoters, while the H-NS protein repressed P_intI1_ but activated both Pc and the functional P2 promoters.

Therefore, the resistance gene cassette expression regulation was considered so far as only relying on Pc strengths and the cassette position effect. These data altogether revealed a much more complex and fine-tuning intertwined regulation, which lies on the perfect counterbalance between cassette recombination mediated by integrase activity and the expression capacity of Pc promoter [26], and on the direct link to host cell physiology process [17,18,19,21].

### 2.7. Integrase Expression in Class 2 Integrons

Although studies focusing on the transcription dynamics of integrase belonging to other integron classes are scarce, some interesting features concerning class 2 integrase regulation had been addressed. Jové et al. [48] had identified that the class 2 integrase promoter sequence P_intI2_ was found 40 bp upstream *intI2* start codon. The *intI2* transcription start site and a potential ribosomal binding site (RBS) were mapped 8 bp and 28 bp downstream this P_intI2_, respectively. It was observed a potential LexA binding site overlapping P_intI2_, suggesting that class 2 integrase activity could also be controlled by the SOS regulon, such as *intI1* and *intIA* from the class 1 integron and the *V. cholerae* CI [17,19]. However, Jové et al. [48] experimentally demonstrated that, in spite of the presence of a LexA binding site in P_intI2_, the *intI2* expression is not regulated by the SOS response. This putative LexA box carried additional polymorphisms in its central region, which may prevent the LexA binding in this site. Moreover, as aforementioned, the functional Pc2 cassette promoters (Pc2A and Pc2B) are found embedded in *attI2*, and not within *intI2*. So, in this case, Pc2 and P_intI2_ are arranged tail-to-tail, in a way that no transcriptional interference would take place. Thus, considering this regulatory scenario, neither LexA nor transcription interference would influence *intI2* transcription, which should be constitutively expressed. Cambray et al. [19] correlated the lack of LexA repressor activity and the presence of inactive IntI enzymes, which probably evolved to avoid the deleterious effects of a constitutively expressed integrase. Therefore, the same correlation can be established for *intI2*, since class 2 integrons normally encode an inactive truncated gene, due to the occurrence of a premature ochre STOP codon (TAA) at position 179 [46]. Conversely, class 2 integrons coding a complete and functional integrase are rarely found, which could be related to a high fitness burden associated with the constitutive expression of *intI2*, since it is devoid of the tight regulation of the SOS system. Additionally, these rare functional class 2 integrons are associated with Pc2 cassette promoter variants (Pc2A-V2 and Pc2B-V2; see above in Section 2.1.2) less efficient in promoting gene cassette transcription. This inverse correlation is the same observed for class 1 integrons, in which weak the Pc variants determine more efficient integrases [26]. Therefore, class 2 integrons are defined by two categories. The most prevalent group carries the Pc2A and Pc2B promoters, which efficiently express a restricted subset of gene cassettes but is incapable of promoting gene cassette innovation due to an inactive integrase. On the other hand, the uncommon category codes a functional integrase that allows novel gene cassette acquisitions but transcribes gene cassettes inefficiently. Based on this evidence, it was proposed that the absence of LexA-mediated *intI2* repression led the selection of mutations that resulted in the integrase inactivation or production of weak Pc configurations, reducing the integron fitness burden [48].

### 2.8. Integrase Expression in Class 3 Integrons

Different from classes 1 and 2 RIs, there are little information concerning the class 3 integron functionality. Although the *intI3* expression had never been experimentally determined as extensively performed for *intI1*, Collis et al. [55] proved that IntI3 was active in recognizing and recombining different gene cassettes within its cognate *attI3* site and at secondary sites in lower frequencies. Indeed, in spite of sharing 59% identity with IntI1 [55], none of the amino acid alterations occurred in the conserved residues of the IntI3 integrase. Moreover, two potential P_intI3_ promoters were identified in the *attI3* site of class 3 integrons [55,56]. However, as found for class 1 integrons, the Pc3 promoter is located within the *intI3* gene (see Section 2.1.3), face-to-face with P_intI3_ promoter [55]. Therefore, transcriptional interference could also take place in class 3 integrons [20,26], in which transcripts starting from Pc could affect *intI3* expression, and hence class 3 integrase recombination activity.

## 3. Post-Transcriptional and Translational Regulation in Integrons

While the transcription regulations of integrase and gene cassettes have been widely approached, there is much less information concerning the translational mechanisms within integrons. It will be discussed below the mechanisms of post-transcriptional and translational control of gene cassettes unraveled, so far, in class 1 integrons, which involve translational coupling, translational attenuation, and regulatory RNAs.

### 3.1. The Role of attI1 Site on Integron-Associated Gene Cassette Translation

The presence of a ribosomal binding site (RBS), known as the Shine–Dalgarno sequence in bacteria (SD sequence), is crucial for the recruitment and assembly of ribosomes and initiation of gene translation. A proper SD sequence usually comprises four bases, normally GGAG, which is complementary to the 16S rRNA small subunit [121].

**Figure 11:**
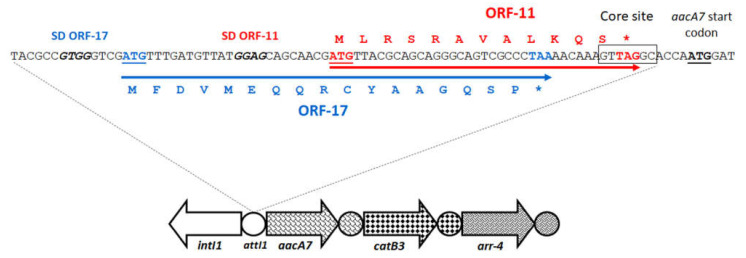
Sequence and organization of the *attI1* site and its regulatory translation signals ORF-11 and ORF-17. The gene cassette array of a class 1 integron is shown (personal communication). The SD sequences of ORF-11 and ORF-17 are in bold italicized caption letters. The start and stop codons of ORF-11 (red) and ORF-17 (blue) are highlighted in bold underlined caption letters. The start codon of the first gene cassette (*aacA7*) is also found in bold underlined caption letters. The 7-bp re-combination core site is boxed. The ORF-11 and ORF-17 overlapped coding sequences are fea-tured by red and blue horizontal arrows, respectively, and their predicted amino acid sequences are indicated. The asterisks represents the translation termination produced by ORF-11 (TAG) and ORF-17 (TAA) stop codons.

The efficient expression of gene cassettes inserted in integrons depends on the presence of the key elements of a canonical translation initiation region (TIR), represented by a functional SD sequence, a plausible start codon, and a proper spacer between them (about 8 bp). Although most of the gene cassettes present a canonical TIR, about 20% of them are devoid of these translational components. In this case, such TIR-deficient gene cassettes may benefit from some regulatory signals found in the *attI1* site when inserted in the first position of class 1 integrons. A small ORF coding 11 amino acids (aa), named ORF-11, is able to enhance the gene expression at the translational level by recruiting ribosomes to TIR-deficient cassettes. The ORF-11 has a canonical TIR, composed of a functional SD sequence (GGAG) placed 8 bp upstream of the ATG start codon. This ORF spans the *attI1* site of class 1 integrons, placed 2–62 bp upstream of the first inserted gene cassette. Since ORF-11 is intrinsically encoded in the *attI1* site, it is present in all transcripts originating from the Pc [122]. However, the ORF-11 overlaps the recombination crossover point of the *attI1* site, in a way that the production of the 11-aa peptide depends on the supply of an in phase stop codon by the first cassette inserted in the array. This stop codon (usually TAG) lies on positions 3 to 5 of the 7 bp GTTRRRY *attC* core site of the inserted cassette (Figure 11). In this way, after ORF-11 translation, the terminated ribosomes might not be released at its stop codon, which leads to a translation restart at initiation codon of the TIR-deficient gene cassette by lateral diffusion of ribosomes through the transcript. Therefore, the translation of ORF-11 supports the expression of the first gene cassette by a translational coupling mechanism, which occurs in polycistronic transcripts where the termination and start codons of contiguous genes are overlapping or close to each other. In fact, the inspection of published sequences supports that the expression of a great proportion of gene cassettes inserted at *attI1* may benefit from translational coupling with ORF-11. Hanau-Berçot et al. [122] also demonstrated that ORF-11 deletion and replacement of its SD sequence or ATG start codon with non-canonical sequences dramatically decreased its translation efficiency. On the other hand, amino acid substitutions in ORF-11 had only a slight effect on translation. Those results demonstrated that the translated ORF-11 itself, and especially its TIR, plays a significant role in enhancing the expression of several TIR-deficient gene cassettes. However, not only TIR-deficient cassettes can be found fused to ORF-11. A remarkable proportion of cassettes with a proper TIR have been found inserted in the first position of integrons coding functional ORF-11 in their *attI1* sites [123]. In this case, an enhanced translation may take place, since both TIRs could augment the recruitment of ribosomes in the vicinity of the gene cassette initiation codon.

A recent study experimentally evaluated the “evolution on demand” of antibiotic resistance in response to selective pressures and integrase activity, and the important role of ORF-11 on antibiotic resistance manifestation was established in that context [124]. It was demonstrated that when the TIR-deficient *aadB* gene cassette was placed in the second position on the integron array, the levels of gentamycin resistance suffered a drastic reduction relative to when the *aadB* was found in the first position. However, this significant difference was not verified when their transcript amounts were evaluated. Once found in the second position, *aadB* transcript amounts decreased in response to cassette position effect, but in a steady and linear fashion, different from what was observed when the level of gentamycin resistance was considered. Such a remarkable difference in gentamycin resistance was explained by the fact that when *aadB* was in the first integron position it was efficiently expressed by translation coupling with the ORF-11, reflecting in high levels of antibiotic resistance. Additionally, this study demonstrated that the several random mutations selected in response to antibiotic pressure led to a heightened translation efficiency of more distal gene cassettes either by incrementing their TIRs or by creating fused cassettes, which also favor translation coupling [124], as discussed below.

Additionally to ORF-11, other ORFs can be created in detrimental of the features of the *attI1* site and inserted gene cassettes. It was observed that another ORF coding 18 aa, named ORF-18, was also involved with TIR-deficient gene cassette translation [125]. The TIR and the first 11 amino acids of ORF-18 were identical to those of ORF-11, but its stop codon was not provided by the recombination core site of the inserted cassette. In this case, the ORF-18 was created upon insertion of the *bla*_OXA-1_ gene cassette, which presents a 5′UTR of 66 bp. Since this cassette does not harbor a potential stop codon in its 7-bp core site, the open reading frame was enlarged in 21 bp into OXA-1 5′UTR (where an in-phase TAA stop codon was found), providing 7 additional amino acids to the short peptide. Therefore, the start codon of the OXA-1 gene was found 45 bp downstream of ORF-18, which contributed to gene cassette expression by the same translation coupling mechanism described for ORF-11 [122]. As found for ORF-11, ORF-18 deletion also abolished OXA-1 translation, which was reset upon the upstream insertion of an SD sequence [15]. The presence of ORF-18 was also detected upstream other TIR-deficient *bla*_OXA-1_-related gene cassettes, such as *bla*_OXA-30_ and *bla*_OXA-31_, and also in *aac(6)-Ib* cassettes encoding acetyltransferases fused to the OXA-1 N-terminal end [15,125], demonstrated the intimate relationship between ORF-18 production and the presence of the OXA-1 5′UTR.

Similarly, a third ORF of 51 bp, encoding a peptide of 17 aa (ORF-17) and harboring a proper TIR, was also intrinsically identified in the *attI1* site [123]. However, unlike ORF-11 and ORF-18, the ORF-17 production does not require the supply of a stop codon by the gene cassette inserted at the *attI1* site. Although ORF-17 overlaps ORF-11, they are encoded in different frames, in a way that ORF-17 starts 26 bp upstream and utilizes an in-frame stop codon inside ORF-11 (Figure 11). The ability of ORF-17 in enhancing translation of downstream gene cassettes was firstly assessed in a class 1 integron harboring the *bla*_GES-1_ inserted in the *attI1* site. However, in this case, *bla*_GES-1_ harbored its own canonical SD sequence. Papagiannitsis et al. [123] experimentally demonstrated that, in spite of the presence of a GES-1 SD sequence, the ORF-11 and ORF-17 were requested to reach highest gene cassette expression levels. It was proposed that the ribosomes gathered in the SD sequences in the 5′UTRs may stabilize transcripts by an in cis mechanism, increasing the GES-1 mRNA life span.

Therefore, it can be assumed that the role of the *attI* site goes clearly beyond its sole involvement in cassette recombination. This region harbors several global regulators binding sites (LexA, cAMP-CRP, H-NS, IHF, FIS) (see Section 2.5) involved with integrase and cassette transcription, and encodes small nonfunctional peptides (ORF-11, ORF-17, ORF-18) that enhance translation of TIR-deficient gene cassettes. Moreover, *attI* sites may up- and downregulate the expression of certain gene cassettes by antibiotic-inducible mechanisms and translation attenuation, respectively. These pieces of evidence altogether highlight the importance of *attI* as gene cassette translational enhancers in integrons.

### 3.2. The Role of attC Site on Integron-Associated Gene Cassette Translation

The cassette position effect clearly interferes with the gene cassette translation efficiencies, and several pieces of evidence pointed to the influence of *attC* sites in this phenomenon and in the differential manifestation of antibiotic resistance. For example, previous studies demonstrated that streptomycin resistance decreased differentially depending on which gene cassette (*aacC1* or *aacA4*) preceded *aadA2* [2]. In addition, *aacA4* was weakly expressed when preceded by *bla*_IMP_ cassettes [35,54]. Jacquier et al. [59] have extensively studied the interference of *attC* sites on downstream cassette translation. They showed that, contrary to what was previously proposed [2], the second structures formed in mRNA due to intra-pairing of the *attC* palindromic region have no influence on transcription termination of downstream gene cassettes. Actually, these stem-loop structures prevent the ribosome movement throughout the mRNA, directly interfering, at the translational level, in the expression of more distal genes regarding Pc [4,59]. It was demonstrated that hairpin destabilization of *attC* showed to enhance the translation of 3′ gene cassettes. Considering that the *attC* sites differ from one another in both sequence and length [6], distinct cassettes (with distinct *attC* sites) would modulate the expression of downstream genes to different extents. In fact, Jacquier et al. [59] observed that gene expression was differentially affected in response to the length and composition of upstream *attC* sites, corroborating previous findings in which the bottom strand of some lengthy *attC* sites presented more complex secondary structures [66]. For example, it had been verified that the levels of *aac(6′)-Ib_7_* translation, the second cassette in the array *bla*_IMP-8_-*aac(6′)-Ib_7_*-*catB4*, varied significantly in response to alterations in stem-loop structures and/or shortening of IMP-8 *attC* site [59].

However, these stem-loop barriers may be relieved by the occurrence of a short ORF within the *attC* site, which would increase the processivity of the translational complex, favoring expression of downstream cassettes via a mechanism of translational termination-reinitiation similar to that described for ORF-11. In termination–reinitiation, the ribosome is not released from the preceding gene cassette stop codon and starts translation at the initiation codon of the short ORF encoded in its *attC* site. This event prevents the stem-loop formation and allows ribosomal scanning along with the transcript, reinitiating translation of the following cassette [59,60,126].

As mentioned above, the expression of fused cassettes occurs by a translation coupling mechanism. Since these fusions are usually formed as a result of total or partial loss of the *attC* site, the lack of stem-loop structures would no longer constitute a physical barrier to ribosome progression, increasing expression of downstream gene cassettes [66]. Fonseca et al. [98] identified the ORF-11 and its TIR at the *attI1* region of a class 1 integron harboring the *gcu14* followed by the fused cassette *bla*_GES-1_/*aacA4*. They verified that transformants harboring this gene cassette array were resistant to β-lactams and aminoglycosides, suggesting that both *bla*_GES-1_ and *aacA4* were expressed. The *gcu14* was a TIR-deficient gene cassette, and they proposed that it was expressed by translation coupling with ORF-11. An SD sequence was found 10 bp upstream of the *bla*_GES-1_/*aacA4* and the lack of stem-loop structures, due to loss of GES-1 *attC* region that led the fusion (Figure 7), probably enhanced the chances of *aacA4* translation. Taking into account that the region involved with the formation of secondary structures was absent in the GES-1 *attC* site, and the role of this site as a translational terminator [59], they suggested that the translation of *bla*_GES-1_ and *aacA4* occurred in a one-step fashion, also by the mechanism of translational coupling [98]. Other studies have reported the occurrence of fused cassettes including *aacA4* (*aac(6′)-Ib*) as the second gene [66], in which it was preceded by a short spacer instead of a complete *attC* site. Therefore, *aacA4* may take advantage of the SD sequence and the start codon of the first gene for its translation, indicating that these genes are favorable for expression in a fused cassette form.

These results altogether indicated that the expression gradient of integron-associated resistance gene cassettes was remarkably affected by the cassette position, in which the number and the nature of upstream cassettes are considered. This phenomenon results from both the relative distance between Pc and the gene cassette and the participation of *attC* secondary structures on translation termination leading to the substantial silencing of downstream gene cassettes [127].

### 3.3. Other Aspects Influencing Translation of Downstream Integron-Associated Gene Cassettes

It was discussed above the role of ORF-11/17/18 in increasing translation of gene cassettes inserted in *attI1* site. However, other regulatory signals may enhance the expression of gene cassettes independent of their position in the integron array. A recent study revealed that expression of the plasmid-borne efflux pump *qepA1*, involved with quinolone resistance, was induced by antibiotic-driven cellular stresses and by regulatory elements found in *qepA1* 5′ end [128]. It was observed that a 250-bp region was found between the class 1 integron *attI1* site and *qepA1.* This region corresponded to the 5′UTR of *dfrB4* gene cassette fused to an unknown *orf*, and this configuration was found to be universal among all *qepA* alleles. Brandis et al. [128] experimentally demonstrated that this region was essential for the efficient expression of *qepA1*, although they were not able to identify which regulatory signals were involved with this regulation. Since efflux pump expression is often tightly regulated to avoid large fitness costs to bacteria, the regulation at the translational level observed for *qepA1* could mitigate such negative effects, restricting high-level expression to environmental conditions in which QepA1 is beneficial.

However, besides *attC* stem-loop structures, other genetic events may negatively interfere with the efficient translation of downstream gene cassettes. A previous study demonstrated how the genetic events that permeate the integron dynamics, such as integration, may decrease gene cassette translation. Hocquet et al. [104] revealed that an *orf* (*gcuF1*) had been inserted immediately upstream *bla*_OXA-28_, which was placed in the second position in a class 1 integron. This event generated a fusion between *gcuF1* and *bla*_OXA-28_, resulting in a polycistronic transcription from Pc, and coupled translation from *gcuF1* TIR. However, such fusion negatively interfered with OXA-28 translation and, consequently, with β-lactam resistance. First, the *gcuF1* presented a weak SD sequence, which led to a decrease in the translation efficiency of the OXA-28. Second, the fusion directly influenced the formation of the OXA-28 signal peptide, interfering with the proper secretion of the oxacillinase to the periplasmic space, which also contributed to low levels of β-lactam resistance. Only upon integrase-mediated excision of *gcuF1* in response to SOS system induction by β-lactams, the *bla*_OXA-28_ could be properly translated and secreted, increasing the levels of β-lactam resistance.

### 3.4. Control of Gene Cassette Expression by Translation Attenuation

**Figure 12:**
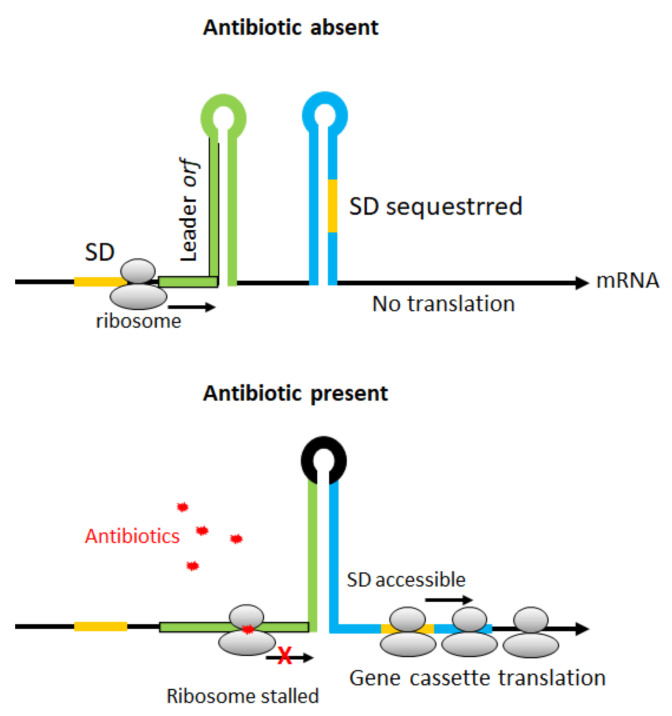
Regulation of antibiotic resistance genes by translation attenuation. The resistance gene presents a leader sequence encoding a small ORF. The translation of the leader ORF leads to the formation of a hairpin structure, inhibiting gene translation by SD sequence arresting (**top panel**). The presence of an antibiotic stalls the ribosome in the leader ORF. This event prompts the production of an additional secondary structure, enabling ribosomes to approach the SD sequence and proceed with resistance gene translation (**bottom panel**).

Integron-associated gene cassettes expression may also be modulated by a mechanism known as translation attenuation. In this post-transcriptional regulatory strategy, the expression of a resistance gene is inducible by its own substrate antibiotic, as found for *ermC* and *cat* genes that are induced by erythromycin and chloramphenicol, respectively [60]. Translation attenuation involves a short leader peptide and the formation of stem-loop structures in transcripts that masks the SD sequence and the initiation codon of the downstream antibiotic resistance gene, preventing its translation (Figure 12). Under the presence of sub-inhibitory concentrations of an inducer antibiotic, the antibiotic-ribosome interactions stimulate the ribosome stalling on a determined site in the short leader peptide, leading to conformational alterations of the mRNA that disrupt the downstream secondary structure, unmasking the SD and start codon of the antibiotic resistance gene, effectively derepressing gene translation [60,126] (Figure 12). The expression modulation by translation attenuation in integrons was firstly described by Stokes and Hall [129]. They demonstrated that the chloramphenicol-resistance *cmlA* gene cassette, placed in the second position of an integron array, harbored transcription elements and translation attenuation signals similar to that found for the inducible *ermC* and *cat* genes [60]. Besides the presence of its own promoter (as discussed in Section 2.3), the large *cmlA* 5′UTR contained a strong SD sequence, an *orf* with a proper TIR encoding a 9-aa leader peptide, and inverted repeat sequences leading to two alternate pairs of stem-loop structures (1 and 2). The leader peptide included a conserved region (Conserved region I) involved with ribosome stalling during peptide translation in the presence of low concentrations of chloramphenicol [60,129]. The stem-loop 2 was involved with sequestration of the downstream SD sequence and initiation codon of the *cmlA* gene. In this way, stalling of ribosomes in the leader peptide region of *cmlA* mRNA contributed to stem-loop 1 disruption, allowing the formation of the alternative stem-loop 2, which disclosed the *cmlA* TIR, allowing ribosome binding and translation of the *cmlA* gene (Figure 12).

Several studies have proposed that the aminoglycoside-inducible translation of *aac* and *aad* genes inserted in class 1 integrons involved the participation of regulatory RNAs, such as riboswitches and small RNAs (SRNAs) (for revision, see references [130,131,132,133,134]). However, it was verified that the DNA sequence coding for the proposed riboswitch RNA actually corresponded to the *attI1* site of class 1 integrons. Moreover, the expression regulation of *aac*/*aad* gene cassettes did not involve a riboswitch, but the ORF-11 peptide production and a mechanism of aminoglycoside-inducible translation attenuation similar to that described above [135].

### 3.5. Integron-Encoded Regulatory RNA

In bacteria, sRNAs act as global regulators of mRNAs, modulating their activity at the posttranscriptional level. A recent study characterized a new mechanism in which in tandem amplification of integron-associated gene cassettes would alter the levels of a putative small RNA (sRNA), which would regulate virulence and global gene expression in *A. baumannii* [134]. These transcripts can be derived from non-coding sequences, such as the UTRs of genes and intergenic regions, and can be independently expressed or produced by the cleavage of longer transcripts catalyzed by RNAses [136].

Some *A. baumannii* strains may switch between the virulent phenotype of opacity (VIR-O) and the avirulent translucent (AV-T), resulting in decreased virulence in vivo [137]. Anderson et al. [134] observed that a duplication of an *aadB*-containing gene cassette array in a composite class 1 integron positively influenced the rates of switching between VIR-O and AV-T cells. However, they noticed that another bacterial subpopulation displaying a third opacity phenotype, designated low-switching opaque (LSO) variant, exhibited a remarkable reduction of switching to AV-T relative to the switching frequency of the VIR-O variants. In this case, a unique copy of *aadB* was identified in the integron of these LSO variants, and hyper-switching to the AV-T state was only observed when the copy number of *aadB* increased. These findings indicated that opacity switching dynamics depended on the *aadB* copy number sRNA. The element regulating this opacity switching was found to be an sRNA of ~300 nucleotides encoded upstream the *aadB* gene cassette, within the 5′CS region of class 1 integrons. Thus, this sRNA would comprise the transcript originating from Pc that included the 5′ end of *intI1*, the *attI1* site, and the short 5′UTR of *aadB* of only 10 bp. In fact, the class 1 integron 5′CS region is known for harboring several regulatory signals, such as global regulator binding sites (LexA, H-NS, IHF, FIS) and the ORF-11 (also ORF-17 and ORF-18) coding segment, which could be involved with the opacity switching regulation. However, site-directed mutagenesis and transposon insertion assays revealed that none of these regulatory elements within the 5′CS appeared to be responsible for controlling switching rates and that this phenomenon was attributed to the production of the proposed sRNA. Despite this, the precise sRNA sequence, how it is generated and processed, its target, and its regulatory mechanism remain to be determined. Therefore, this finding is clear evidence of the versatility of integrons, which includes functions other than the acquisition and expression of antibiotic resistance gene cassettes.

## 4. Conclusions

Here, we compiled information regarding the different aspects that permeate the expression in integrons, highlighting the several delicately controlled transcriptional and translational strategies that ultimately reduce the fitness burden and provide an additional selective advantage to bacterial hosts in challenging environments. Such intriguing genetic platforms are subjected to an elegant, clever, and fine-tuning regulation that involves a powerful balance between expression and recombination of gene cassettes, and an intricate interplay with the cell physiology components and environmental stimuli, being the basis of the adaptation on demand involved with the short-term evolution of integrons. Therefore, this elaborate regulatory network reveals that the integron dynamics are more tricky than previously thought, proving that these versatile elements are not only gene cassette acquisition and expression systems, but have a wider role in creating bacterial diversity.

## Figures and Tables

**Table 1:** Main differences among resistance/mobile and chromosomal integrons.

Integron Features	Resistance/Mobile Integrons (Example of Class 1 Integron)	Chromosomal Integrons (Example of IntVchA)
Genetic context	Mobile genetic elements (transposons, plasmids, ICEs)	Chromosome
Integrase gene	*intI1*	*intIA* (*intI4*)
Number of gene cassettes	Dozens	Hundreds
Nature of gene cassettes	Antibiotic resistance	Unknown functions, metabolic functions, antibiotic resistance (less frequent)
Cassette recombination sites	*attC* (variable in sequence and size)	VCR (~120-bp repeat)

**Table 3:** Genetic and functional features of Pc2 variants in class 2 integrons.

Pc2 Promoter Variants	–35 Sequence	Spacer Region (bp)	–10 Sequence	Position	Transcriptional Activity	Reference
Pc2A Pc2A-V2	TTTTAA TTTTAA	17 17	TAAAAT TAAAGT	*attI2*	YES	[48,49]
Pc2B PC2B-V2	TTGTAT TTATAT	16 16	TTTAAT TTTAAT	*attI2*	YES	[48]
Pc2C	GTGACA	19	TAAAAT	*attI2*	YES	[48]
Pc2D	TTGCAA	18	TATTCT	*attI2*	NO	[48]
Pc2E	TGGCTA	17	(TGN)TAAGCT	*intI2*	NO	[48]
